# Novel transcripts reveal a complex structure of the human *TRKA* gene and imply the presence of multiple protein isoforms

**DOI:** 10.1186/s12868-015-0215-x

**Published:** 2015-11-18

**Authors:** Kristi Luberg, Rahel Park, Elina Aleksejeva, Tõnis Timmusk

**Affiliations:** Department of Gene Technology, Tallinn University of Technology, Akadeemia tee 15, 12618 Tallinn, Estonia; Competence Center for Cancer Research, Tallinn, Estonia; VIB lab for Systems Biology & CMPG Lab for Genetics and Genomics, Leuven, Belgium; French National Institute for Agricultural Research, Paris, France

**Keywords:** TRKA, NTRK1, 5′ RACE, Glycosylation, Nuclear localization, Isoforms, Alternative splicing

## Abstract

**Background:**

Tropomyosin-related kinase A (TRKA) is a nerve growth factor (NGF) receptor that belongs to the tyrosine kinase receptor family. It is critical for the correct development of many types of neurons including pain-mediating sensory neurons and also controls proliferation, differentiation and survival of many neuronal and non-neuronal cells. *TRKA* (also known as *NTRK1*) gene is a target of alternative splicing which can result in several different protein isoforms. Presently, three human isoforms (TRKAI, TRKAII and TRKAIII) and two rat isoforms (TRKA L0 and TRKA L1) have been described.

**Results:**

We show here that human *TRKA* gene is overlapped by two genes and spans 67 kb—almost three times the size that has been previously described. Numerous transcription initiation sites from eight different 5′ exons and a sophisticated splicing pattern among exons encoding the extracellular part of TRKA receptor indicate that there might be a large variety of alternative protein isoforms. *TrkA* genes in rat and mouse appear to be considerably shorter, are not overlapped by other genes and display more straightforward splicing patterns. We describe the expression profile of alternatively spliced *TRKA* transcripts in different tissues of human, rat and mouse, as well as analyze putative endogenous TRKA protein isoforms in human SH-SY5Y and rat PC12 cells. We also characterize a selection of novel putative protein isoforms by portraying their phosphorylation, glycosylation and intracellular localization patterns. Our findings show that an isoform comprising mainly of TRKA kinase domain is capable of entering the nucleus.

**Conclusions:**

Results obtained in this study refer to the existence of a multitude of *TRKA* mRNA and protein isoforms, with some putative proteins possessing very distinct properties.

**Electronic supplementary material:**

The online version of this article (doi:10.1186/s12868-015-0215-x) contains supplementary material, which is available to authorized users.

## Background

Tropomyosin-related kinase A (*TRKA*, official name neurotrophic tyrosine kinase, receptor, type 1, or *NTRK1*) gene encodes the high affinity receptor for a neurotrophin nerve growth factor (NGF). TRKA with the highly similar receptors TRKB and TRKC belongs to the group of tyrosine kinase receptors. TRKB binds neurotrophins brain-derived neurotrophic factor (BDNF) and neurotrophin-4 (NT-4) while TRKC is the predominant receptor for neurotrophin NT-3, although TRKA and TRKB can also be activated by NT-3 [1]. Signaling initiated by the NGF-TRKA complex is crucial for the development of pain-mediating sensory neurons [2], postganglionic sympathetic neurons [3] and basal forebrain cholinergic neurons [4]. NGF also affects cells of non-neuronal tissues, such as epithelial and smooth muscle cells, and is very important in thymic tissues [5].

Neurotrophin binding to the TRK extracellular domain leads to receptor’s dimerization, activation of its intrinsic kinase activity and autophosphorylation. Subsequently, the signaling pathways similar for all the TRK receptors are activated. These include the rat sarcoma/mitogen-activated protein kinase (Ras-MAPK), phosphatidyl-inositol 3 kinase (PI3K) and phospholipase C-γ1 (PLC-γ1) pathways to promote survival and differentiation, and adjust synaptic plasticity [6–8].

In addition, several neurotrophin independent signaling events have been described, including transactivation of receptor tyrosine kinases by adenosine 2A receptors [9, 10], pituitary adenylate cyclase-activating polypeptide receptor [11], low-density lipoprotein receptor-related protein 1 [12], epidermal growth factor receptor [13] and antidepressants [14].

**Fig. 1:**
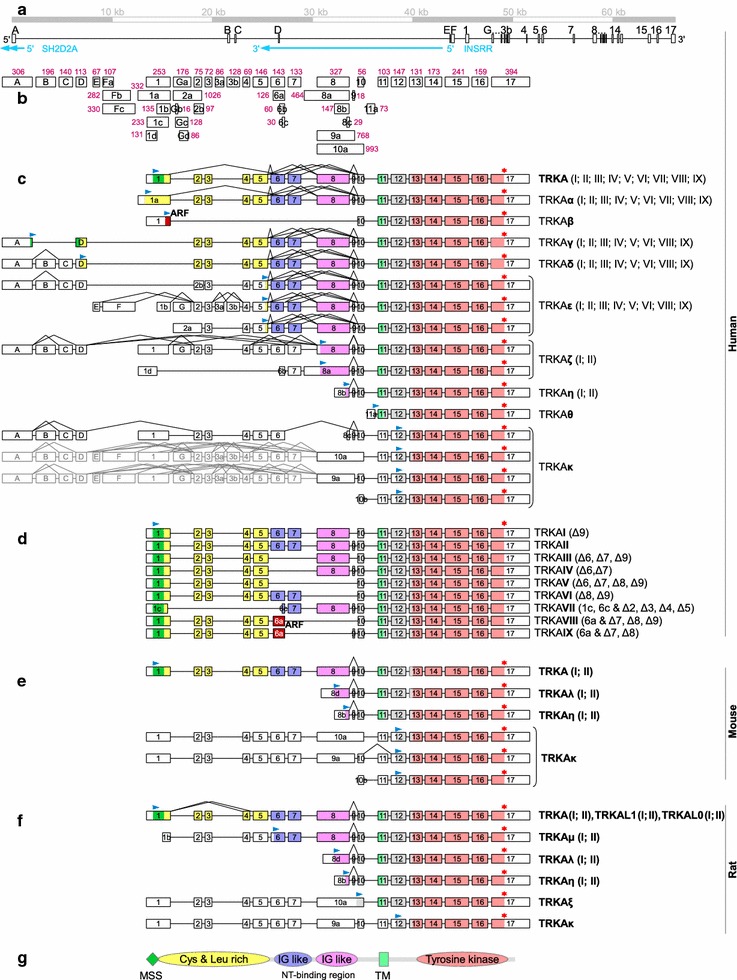
Human *TRKA* gene locus and predicted protein isoforms of human, rat and mouse TRKA. Exons are illustrated as *boxes* and introns as *lines*. Exons are drawn to scale in **a**–**f** and introns in **a**. **a** Chromosomal organization of the human *TRKA* gene. Also shown are two genes encoded from the opposite strand and partially (*SH2D2A*) or entirely (*INSRR*) overlapping the *TRKA* gene. **b** All human *TRKA* exons with size in bp-s shown in *dark pink*. Representational scheme of *TRKA* alternative transcripts and putative protein isoforms of human (**c** and **d**), mouse (**e**) and rat (**f**) origin, based on the results of 5′ RACE, RT-PCR study and in silico analysis. The names of encoded proteins are noted on the right. Alternative N-termini as compared to the conventional TRKAII isoform are labeled as α, β, γ, δ, ε, ζ, η, θ, κ, λ, μ and ξ. Only one ORF per transcript has been marked with start site (*blue arrowhead*) and stop codon (*red asterisk*). *Roman numerals* designate protein isoforms missing various parts of the extracellular region as illustrated for TRKAI…TRKAIX **d**. Exons 10a and 9a can either serve as 5′ exons or be the products of intron retention (due to this, the block of exons upstream of exons 10a and 9a is illustrated in *gray*). *ARF* alternative reading-frame. The L0 isoform of rat TRKA is produced from transcripts that do not include exons 2–4 and L1 from mRNAs missing exons 2 and 3. **g** the protein domains of TRKAII (*MSS* membrane signal sequence, *Cys and Leu rich region* cysteine and leucine rich region, *IG* *like* immunoglobulin-like domain, *TM* transmembrane)

Human *TRKA* gene is located on chromosome 1 and has been described to span 23 kb. Seventeen exons (named 1…17; Fig. 1), that are relatively well conserved in rat and mouse as compared to human *TRKA* gene, [the basic local alignment search tool (BLAST) algorithm gives 85 % of similarity in both cases] have been characterized [15–17].

The extracellular portion of TRKA receptor, coded by exons 1–10, is responsible for ligand binding and is subjected to post-translational glycosylation. A sequence coded by exon 1 directs the receptor to the cell membrane. The predominant part of the extracellular region constitutes of the first and second immunoglobulin-like (Ig-like) domains coded by exons 6…8, of which the second is directly in contact with NGF [18]. The transmembrane domain of TRKA is encoded by exon 11 and the intracellular tyrosine kinase domain by exons 13…17 [19, 20].

The glycosylation of the receptor’s extracellular segment plays an important role in signaling and localization of the protein. There are four N-glycosylation sites that are highly conserved within the TRK family and at least five more variable sites that are used in TRKA. The lack of glycosylation results in autophosphorylation and constitutive kinase activity of the core protein as well as incapability to be directed to the cell membrane [21, 22].

*TRKA* gene is a target of alternative splicing which can result in several different protein isoforms. At the moment, only three human isoforms (TRKAI, TRKAII and TRKAIII) and two additional rat isoforms (TRKA L0 and TRKA L1) have been described. TRKAII is the full-length isoform. In mRNA encoding TRKAI an 18 bp exon 9 has been spliced out resulting in a protein lacking 6 aa in the juxtamembrane region of the TRKA receptor. The full-length TRKAII is mainly expressed in neuronal tissues and TRKAI in non-neuronal tissues. They appear to have no relevant difference in binding to NGF [23]. In contrast, binding to NT-3 is significantly stronger in the case of TRKAII compared to TRKAI [24]. The third alternatively spliced transcript *TRKAIII* is lacking exons 6, 7 and 9. This results in the absence of the first immunoglobulin-like domain and several N-glycosylation sites. As a consequence, TRKAIII is not able to bind NGF and is instead constantly autophosphorylated and activated. Alternative splicing of *TRKA* mRNA to generate the isoform TRKAIII is upregulated by hypoxia. TRKAIII is expressed in undifferentiated early neural progenitors, in a subset of neural crest-derived tumors (notably in neuroblastomas) and in thymus, a physiologically hypoxic organ [25, 26]. Unlike TRKAI/II, this isoform is not inserted in the plasma membrane, but is retained in endoplasmatic reticulum (ER) and Golgi complex and promotes genetic instability [27]. In rats, similarly to humans, exon nine can be spliced out [23]. In addition, rat splice variants termed TRKAL1 and TRKAL0 lack respectively either two of the leucine-rich motifs or all three [28]. Isoforms of TRKA in mouse have not yet been described.

A genetic disorder congenital insensitivity to pain with anhidrosis (CIPA), also called hereditary sensory and autonomic neuropathy type IV, has various symptoms such as absence of reaction to noxious stimuli (insensitivity to pain), anhidrosis (inability to sweat), self-mutilating behavior and mental retardation. CIPA is caused by non-functional or absent TRKA receptor due to mutations in *TRKA* gene [29]. On the other hand, excessive NGF-TRKA signaling hypersensitizes pain-mediating neurons resulting in chronic pain [30] or causes allergic skin inflammation [29], hyper-responsiveness of airway epithelial cells and/or aberrant activation of sensory neurons, implicated in acute conditions such as asthma [31].

Alterations in *TRKA* expression or mutations in the gene have been detected in several tumors. TRKA was discovered as an oncogene in colon carcinoma fused with tropomyosin gene [32]. Genomic DNA rearrangements of TRK genes can influence carcinogenic progression in non-neuronal tissues such as breast cancer [33], papillary thyroid carcinoma [34] and medullary thyroid carcinoma [35]. In neuroblastomas, *TRKA* upregulation is seen in tumors with good prognosis, while TRKB is up-regulated in unfavorable and aggressive tumors [36]. However, TRKA can also be involved in the late stages of cancer progression by promoting stress-resistance and neovascularization—for example in neuroblastomas by TRKAIII isoform [37]. During the progression of Alzheimer’s disease, all TRK receptors are down-regulated in cholinergic neurons of nucleus basalis, a brain region which dysfunction is associated with cognitive decline in Alzheimer’s disease [38]. The cholinergic neurons depend on NGF which is synthesized by the target cells within the hippocampus and cortex. *TRKA* expression is also decreased in the parietal cortex of patients with Alzheimer’s disease [39, 40]. Moreover, the withdrawal of NGF in differentiated rat pheochromocytoma PC12 cells initiates the accumulation of beta-amyloid protein and is followed by apoptotic death [41].

In this study, we show that the *TRKA* gene in rat, mouse and especially human is more complex than previously thought. We also show that the human *TRKA* has multiple 5′ terminal exons and an intricate splicing pattern involving exons that encode the extracellular part of TRKA protein. It can be theorized that these novel *TRKA* transcripts encode numerous TRKA protein isoforms which have not yet been characterized.

## Results

### An elaborate arrangement of the human *TRKA* gene revealed by novel transcription initiation sites

In silico analysis of the human *TRKA* gene structure using UCSC genome browser [42] to align the *TRKA* mRNAs and expressed sequence tags (ESTs) from GenBank to the genomic sequence indicated a higher level of variability among *TRKA* transcripts and a longer span of the gene than previously described in the literature. For this reason, we performed reverse transcription polymerase chain reaction (RT-PCR) and rapid amplification of 5′ complementary DNA ends (5′ RACE) analyses to better describe *TRKA* gene and its transcripts. In the RT-PCR study, we used a selection of adult and fetal tissues and different regions of the nervous system. Also included were neuroblastoma cell-lines SH-SY-5Y and SK-NMC which are known to express *TRKA*. Total RNA from SH-SY-5Y neuroblastoma cells and thalamus were used in 5′ RACE experiments as these tissues showed relatively high levels of *TRKA* expression in our preliminary experiments. From the information obtained of mRNAs, we predicted potential TRKA protein isoforms and named them in this study as follows: isoforms with different N-termini from the conventional TRKAII are named as α, β, γ, δ, ε, ζ, η, θ and κ (Fig. 1c), isoforms which lack different parts in their extracellular portion are distinguished with roman numerals I…IX (Fig. 1d; protein sequences are listed in Additional file 1).

**Fig. 2:**
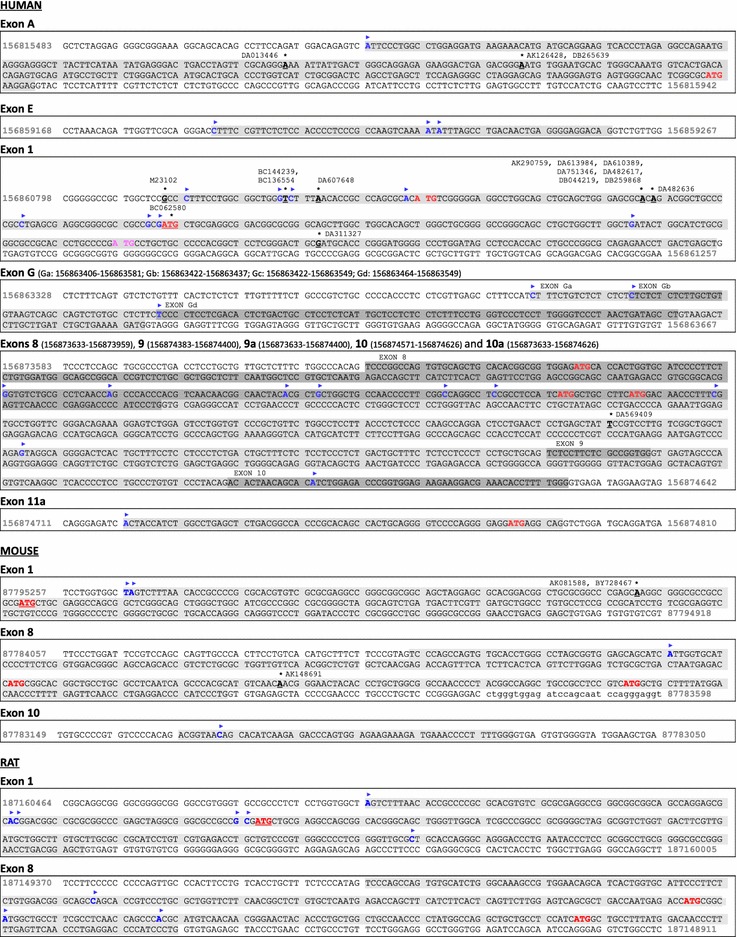
The human *TRKA* gene has numerous transcription start sites producing more alternative 5′ exons than mouse and rat *TrkA* genes. Results of the 5′ RACE analysis of *TRKA *mRNA of human (combined results of extracts from thalamus and SH-SY-5Y cell line), mouse (brain at embryonic day 13) and rat (PC12 cell line). Novel transcription start-sites are indicated in *blue bold letters* and marked with a *blue arrow head*. Transcription initiation sites deduced from GenBank sequences of previously described mRNAs and ESTs that are obtained with 5′ RACE analysis are displayed in *bold underlined letters* and designated with *black asterisks* with the corresponding GenBank accession numbers shown. *Red and bold letters* indicate alternative putative translation start sites with the conventional translation initiation site (producing TRKAII protein isoform) *underlined*. The translational start site from alternative reading frame that is used if exons 2…9 are spliced out in human tissues between exons 1 and 10 is indicated in *pink bold letters*. Exons are marked with *grey* background. The numbering is based on the human genome assembly GRCh38 (hg38), the mouse genome assembly GRCm38 (mm10) or the rat genome assembly RGSC6.0 (rn6)

We detected multiple transcription start-sites in exon 1, most of which are located upstream of the conventional translation start-site in the position 156860935 nt of the GRCh38 human genome assembly (Fig. 2). However, there is an additional in-frame ATG in the position 156860857 nt that was included in some forms of exon 1 (named in this study as 1a). If this AUG is sterically accessible for the ribosome, an N-terminally elongated protein (named here as TRKAα) compared to the conventional TRKAII would be produced. On the other hand, some transcripts had a shorter exon 1 (exon 1b) with no in-frame AUG codons. Translation from mRNAs with exon 1b probably starts from the next in-frame AUG which is in exon 5 (in genomic position 156868231 nt) creating TRKAε. The online transmembrane topology and signal peptide predictor Phobius [43] predicts no membrane signal sequences for TRKAα or TRKAε.

Analysis of GenBank sequences [GenBank:DA013446, GenBank:DB265639, GenBank:AK126428] revealed the presence of a novel 5′ terminus of *TRKA* mRNAs formed by alternative transcription initiation and usage of four novel exons that are in this study termed as A, B, C and D (Fig. 1). We confirmed with 5′ RACE using mRNAs from adult human thalamus that exon A serves as a transcriptional start-site (Fig. 2). Exon A is located approximately 45 kb upstream of exon 1 and therefore the size of *TRKA* gene is 67 kb—almost three times bigger than previously thought. It is of interest to note that exon A is in the intron between the second and the third exon of sarcoma protein homology 2 domain protein 2A (*SH2D2A*) gene that is encoded on the complementary strand from *TRKA*. The transcription start sites of these genes are in a head-to-head orientation and lie less than 1 kb apart. On the same strand and upstream from *SH2D2A* gene, there is insulin receptor-related protein (*INSRR*) gene which is completely embedded into *TRKA* gene (Fig. 1a). Thus, *TRKA* is overlapped by two genes and that is rather unusual according to Veeramachaneni and associates, who identified only 18 overlapping gene trios in the human genome [44]. Transcription start sites of *TRKA* exon 1 and *INSRR* gene are in a nearby head-to-head orientation and are less than 2 kb apart. *TRKA* exon D and a coding exon in *INSRR* gene overlap by more than 80 bp.

Translation of transcripts with exons A and D followed by exons 2, 3, etc., starts most probably from an AUG near the end of exon A (from the genomic position 156815830 nt) producing TRKAγ. Besides this major ORF, these transcripts also have many small upstream ORFs (uORFs) in different reading-frames that are no bigger than 111 nt. For mRNAs with exons A-C-D or A-B-C-D, the primary ORF starts in exon D (from genomic position 156842144 nt) generating TRKAδ. uORFs of these transcripts are longer, reaching 336 nt. Phobius predicts membrane signal sequence for TRKAγ but not for TRKAδ.

**Fig. 3:**
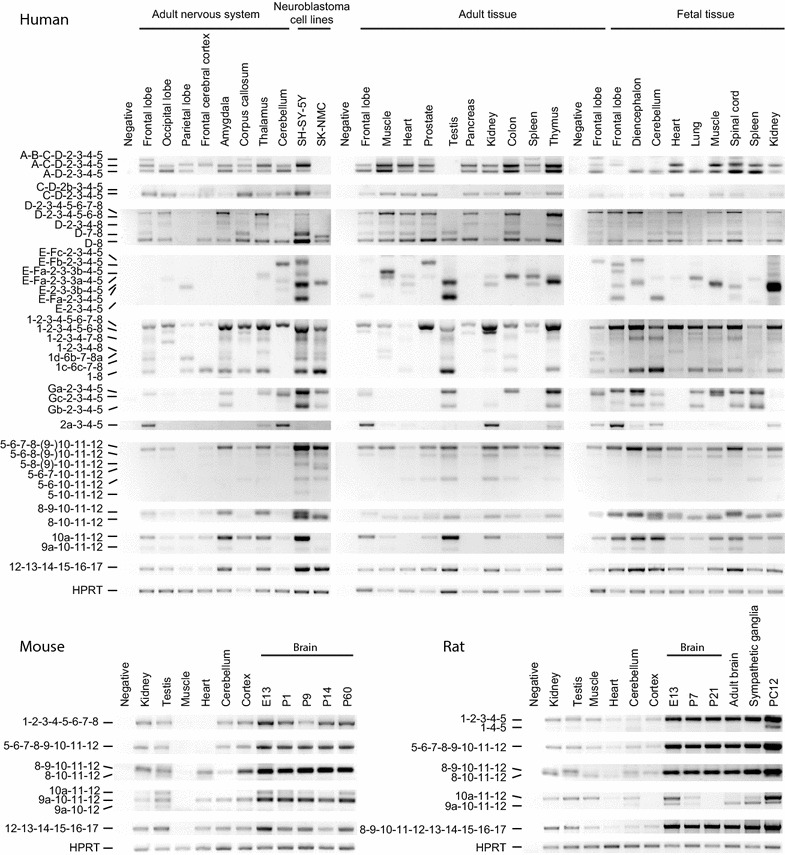
Semi-quantitative analysis of *TRKA* transcripts by RT-PCR in different human, mouse and rat tissues, in human neuroblastoma SH-SY5Y and SK-NMC cells and in rat pheochromocytoma PC12 cells. The exons present in the PCR products are marked on the *left*. Hypoxanthine guanine phosphoribosyltransferase (*HPRT*) gene specific primers were used for the housekeeping control. *E13* samples from brain at embryonic day 13, *P1, P7, P9, P14, P21 and P60* samples from brain at postnatal days 1, 7, 9, 14, 21 and 60, respectively

Using 5′ RACE analysis, we also identified novel exons E, G and 11a as alternative *TRKA* 5′ exons (Fig. 2). Exon E is located 1.5 kb upstream of exon 1 and may be followed by novel exon F which has three alternative 3′ termini (generating Fa, Fb and Fc exon variants). Exon E is even closer to the *INSRR* gene than exon 1, but does not overlap with it. RT-PCR also revealed two novel exons located downstream of exon 3 that are named in this study as 3a and 3b that can be spliced into mRNAs with 5′ exons E and Fa (Figs. 1b, 3). Exon G is located between exons 1 and 2, specifically 2.3 kb downstream of exon 1, and has alternative versions Ga, Gb, Gc and Gd that differ from each other in both 5′ and 3′ splice sites (Fig. 2). Using RT-PCR we also detected intron retention between exons G and 2 generating an extended exon that is here named as 2a. The major ORF of mRNAs with exons E, F, G and 2a is located to exons 5…17 and encodes TRKAε. All of these mRNAs have 1…3 uORFs in alternative reading frames that are less than 168 nt, with the exception of transcripts that start with exon 2a in which case there are 18 uORFs with a maximum length of 321 nt.

The 5′ exon 11a is located 95 bp downstream of exon 10 and transcripts with this exon encode for TRKAθ which has no predicted membrane signal sequence. The first ATG is located near the 3′ end of exon 11a at genomic position 156874785 nt. There are no uORFs on these mRNAs.

The exon complexes A…D and E–F and exons 1, G, 2a and 11a are all mutually exclusive as we didn’t observe any transcripts with different combinations of these 5′ exons or exon complexes.

Surprisingly, we also detected transcription initiation from exon 10 and from many different nucleotides within exon 8 (Fig. 2). Most of the mRNAs that have exon 8 as the 5′ exon contain in-frame AUG codons present near the 3′ end of exon 8 (this variant of the exon is named in here as 8b). Assuming that the first of these codons (at genomic position 156873905 nt) serves as a translational start-site, the protein produced is TRKAη. These transcripts have no uORFs. *TRKA* transcripts that start with exon 8c that is shorter than 8b, which contains no in-frame AUG codons, or with 10b have a major ORF situated in exons 12…17 with the first ATG in genomic position 156875555 nt and encoding TRKAκ protein isoform. 2 uORFs with a maximum length of 171 nt are also present.

According to one EST and our 5′ RACE results, transcription can also start in the intron between exons 8 and 9, from exon 10a which contains exons 9 and 10 and the intron between them (Fig. 2). With RT-PCR we also detected the presence of exon 9a that is similar to exon 10a, but its 3′ end coincides with the one in exon 9 (the intron between exons 9 and 10 is spliced out in this case). With RT-PCR (data not shown) we detected the presence of exons 9a and 10a in transcripts that have additional exons in 5′ direction and in that case exons 9a and 10a are the products of intron retention as they span from the 5′ end of exon 8 to the 3′ end of exon 9 or 10, respectively. In either case, there are multiple ORFs in these transcripts, but the ORF that extends into the 3′ exons and which translation would salvage these mRNAs from nonsense mediated decay (NMD), encodes for TRKAκ protein isoform. However, the true functionality of these transcripts is presently unknown.

### Human *TRKA* exons 2…9 encoding the extracellular domain are all cassette exons with an intricate splicing pattern

Several exons of the human *TRKA* gene can be spliced out. All of these encode parts of the extracellular domain of TRKA protein. The splicing of cassette exon 9 has been described and the protein isoform encoded by exons 1…17 is known as TRKAII and the exclusion of amino acids encoded by exon 9 leads to the production TRKAI protein isoform [23]. Formerly, exclusion of exons 6, 7 and 9 from *TRKA* mRNA has also been reported, giving rise to a protein isoform TRKAIII [25]. We detected alternative splicing of all exons encoding the extracellular domain, with the only exception of exon 10 (Figs. 1c, d, 3). Additionally to the mRNA isoforms described previously, alternative splicing that does not disturb the reading frame of transcripts was detected as the exclusion of exon complexes 6-7 (producing TRKAIV), 6-7-8-9 (TRKAV), 8-9 (TRKAVI) and 2-3-4-5. In the latter case, alternative splice sites are used for exons 1 and 6 resulting in exons 1c (20 nt shorter from the conventional exon 1 from the 3′ end) and 6c (113 nt shorter from exon 6 from the 5′ end). The protein encoded by transcripts with exons 1c-6c-7…17 is TRKAVII. Theoretically, the same splicing patterns can also be present in transcripts with other 5′ termini and thus, the putative protein isoforms of human TRKA also include TRKAαI, TRKAαII, TRKAγI, etc.

Moreover, there are many splice forms that produce a frame-shift. This includes transcripts with exons 6a-10 and 6a-9-10 and lacking exons 7 and 8 (and 9 in the former case; Fig. 1c) as identified with 5′ RACE analysis. Exon 6a is a shorter version of exon 6 with 17 nt missing from the 5′ end as compared to exon 6. The sequence of exon 6a is read in an alternative reading frame, producing isoforms TRKAVIII and TRKAIX, respectively. Alternative reading-frame of exon 1 is most probably used with transcripts which are missing the complex of exons 2…9, generating TRKAβ which lacks membrane signal sequence according to Phobius program. Translational start-site most probably used in this case is located 100 nt from the 3′ end of exon 1 (at genomic position 156861047 nt). One uORF of 294 nt is present in that mRNA.

One of the many types of human *TRKA* transcripts identified with RT-PCR contained exons 1d-6b-7-8a with exons 2…5 spliced out. Exon 1d is 122 nt shorter from exon 1 from the 3′ terminus, exon 6b is 83 nt shorter from exon 6 from the 5′ end, and 8a is an extended form of exon 8 with additional 137 nt in its 5′ terminus. This mRNA most probably encodes for TRKAζII with the translational initiation site in exon 8 (at genomic position 156873668 nt) although it has 4 uORFs with the maximum length of 297 nt. TRKAζII possesses no predicted membrane signal sequences.

With RT-PCR we identified the exclusion of exon 7 or exon complexes 2…6, 2…7, 5-6, 5…7, and 7…9 that also produce frame-shifts. To escape the NMD pathway, the ORF expressed from these transcripts should encode TRKAκ (in case exons 7-8-9 are spliced out) or TRKAζI/TRKAζII (in case of other splice-combinations). However, all these mRNAs contain uORFs that are in some cases rather large. Therefore, the exact function (if any) of these transcripts is uncertain.

With primers located in *TRKA* exons C and 5 two PCR products with similar lengths (cDNA from frontal cerebral cortex, Fig. 3) were identified with sequencing. It appeared that the longer product contained an extended form of exon 2 with additional 22 bp in its 3′ end (that we named 2b) compared to the normal exon 2. This induces a frameshift and results in a premature stop codon. To escape the NMD pathway, the protein translated from that transcript should be TRKAε, regardless of multiple uORFs with maximum length of 189 nt.

*TrkA* alternative transcripts lacking exons 2…3 (*TrkAL1*) or exons 2…4 (*TrkAL0*) have been found in Wistar rats [28]. However, we did not detect such transcripts of human *TRKA* gene, suggesting that these transcripts might be rodent-specific.

### Expression pattern of* TRKA* transcripts in human tissues and cell-lines

Next, we wanted to elucidate the expression pattern of *TRKA* transcripts in different tissues. To this purpose, we examined *TRKA* transcript expression with RT-PCR analysis in a selection of human tissues, brain regions and two neuroblastoma cell-lines. The results (Fig. 3) indicated that transcripts with 5′ exons A-D were expressed in most examined tissues, except in adult testis and frontal cerebral cortex as well as fetal frontal lobe. Expression of this transcript was also seen in neuroblastoma SH-SY5Y cells, but not in neuroblastoma SK-NMC cells. Transcripts with exons A-C-D were widely expressed in different tissues apart from adult testis, fetal diencephalon, cerebellum and lung, and SK-NMC cells. The rare splice variant with exons A-B-C-D was expressed at very low levels in several tissues, more significantly in adult frontal lobe, corpus callosum, and spleen as well as in fetal spinal cord. The expression of exon 2b was even rarer, as we detected it only in frontal cerebral cortex.

The transcript including exons D and 8, and excluding exons 2…7 had a wide expression pattern with the exception of adult parietal lobe and with relatively higher expression in SH-SY-5Y cells and adult prostate and thymus. The expression of mRNAs with exons D-7-8 with an absence of exons 2…6 was not unanimous and displayed higher levels in adult corpus callosum, muscle, heart, testis and colon, fetal cerebellum, heart and spinal cord, and SH-SY-5Y cell line. Human *TRKA* transcripts with exons D-2-3-4-5-6-8 (missing exon 7) and D-2-3-4-8 (missing exons 5-6-7) were expressed in low levels in all studied tissues except adult parietal lobe and the SK-NMC cell line. We did not observe mRNAs with exons D-2-3-4-5-6-7-8 in adult cerebellum, testis and spleen, fetal lung and SK-NMC cells. The highest expression of these mRNAs was found in adult amygdala, thalamus, muscle and thymus.

Exon complex 1…8 was detected in mRNAs from all tissues studied, with especially high levels in adult amygdala, thalamus, prostate, kidney and thymus, fetal diencephalon, heart and spinal cord and SH-SY-5Y cells. mRNAs with exons 1c-6c-7-8 were detected in low levels in adult frontal and parietal lobe, amygdala, muscle, testis and colon and neuroblastomas. Relatively low levels of transcripts with exons 1-2-3-4-5-6-8 (with exon 7 spliced out), 1-2-3-4-7-8, 1-2-3-4-8 and/or 1d-6b-7-8a that are all encoding the TRKA ζ isoform, were observed in all tissues analyzed with the exception of adult parietal lobe, frontal cerebral cortex and cerebellum. The expression of transcripts with exon 1 and 8 (missing the cassette of exons 2…7) was highest in SK-NMC cells, testis and fetal cerebellum, and below detection limit in adult occipital lobe, cerebellum, muscle, prostate, pancreas, kidney and spleen.

Novel 5′ exons E, F and G were expressed in many tissues with the highest levels in cerebellum, neuroblastomas, testis, colon, thymus and fetal frontal lobe, diencephalon, lung, muscle and spinal cord. Adult frontal cerebral cortex, corpus callosum, pancreas and fetal heart were the tissues where expression of these exons was below the detection limit. Exon 2a was expressed in only a small selection of tissues, most notably in frontal lobe, cerebellum and kidney.

Splicing in the region of exons 6…8 was a rare event with the highest prevalence of transcripts lacking exon 7 or exons 7…9. Splice forms where exons 8-9, 6-7-8-9 or 6-7 are spliced out appeared to be neuroblastoma specific, as they were detected only in the samples from SH-SY5Y and SK-NMC cells. These mRNAs encode TRKA proteins without one or two Ig-like domains.

The transcript containing exon 9 is predominately expressed in neural tissues while the exclusion of exon 9 has been observed in peripheral tissues [23, 24]. In that respect, the results of this study are in accordance with previous findings. In some tissues, such as adult prostate, fetal diencephalon and heart as well as in neuroblastoma SH-SY5Y, both of the transcripts were detected.

Transcripts with exon 10a were detected in many tissues with the exceptions of SK-NMC cells, adult heart, pancreas and spleen. The novel exon 9a was expressed at minute levels in frontal lobe, occipital lobe, amygdala, thalamus, muscle, testis, fetal diencephalon and spinal cord and neuroblastoma cells.

We failed to design PCR primers with which it would be possible to amplify *TRKA* sequences with exon 11a. Most probably the expression level of these transcripts is very low.

All analyzed *TRKA* transcript variants shared common 3′ exons as we did not detect any alternative splicing after exon 11. Therefore, the expression pattern of exons 12…17 corresponded to *TRKA* mRNA overall levels and was the highest in adult amygdala, thalamus, testis, neuroblastomas and in fetal diencephalon and spinal cord according to PCR results with primers targeting exon 12 and 17 (Fig. 3).

### Mouse and rat* TrkA* mRNAs display smaller variability than human* TRKA* transcripts

To examine whether the complex splicing pattern seen in human tissues is conserved in other mammals, mouse and rat samples were also analyzed. Rat and mouse tissues to be examined were chosen according to previous results in human and taking into consideration the tissue and/or mRNA availability. Rat PC12 cell line was included in the rat expression panel as it has been shown to express high levels of *TrkA* and has been the major tool to perform research on TRKA receptor, including this study [45].

Total RNA isolated from mouse brain E13 tissue and rat PC12 cells was used in 5′ RACE experiments as these tissues showed relatively high levels of *TrkA* expression. Our 5′ RACE results and a few mouse sequences in public databases indicated that most often transcription is initiated from exon 1 upstream of the conventional translation-start site at position 87795144 of the GRCm38 mouse genome assembly and at 187160312 of the RGSC6.0 rat genome assembly (Fig. 2). However, we detected rat *TrkA* mRNAs that have 5′ end in exon 1, but downstream of this AUG. This variant of exon 1 is named here as 1b and the major ORF of rat *TrkA* transcripts containing exon 1b starts from exon 6 at genomic position 187153794 and encodes TRKAμ protein (Fig. 1f). Three small uORFs with length up to 63 nt are also present.

A sequence in GenBank [GenBank:AK148691], that has been obtained by 5′ RACE, and also sequences from our 5′ RACE results additionally characterize both mouse and rat *TrkAs* with 5′ termini inside exon 8 (named in this case as 8d or 8b, for longer and shorter versions, respectively). These transcripts encode TRKAλ and TRKAη proteins, both of which have translation-initiation sites in exon 8: TRKAλ at genomic position 87783826 (mouse) or 187149147 (rat), and TRKAη at position 87783724 (mouse) or 187149045 (rat). Mouse and rat transcripts with 5′ exons 8d and 8b have uORFs up to 282 nt, with the exception of mouse mRNAs with exon 8b, which have no uORFs.

Similarly to human mRNA, we also identified transcription initiation from within *TrkA* exon 10 (named in that case as 10b) in mouse mRNA, but not in rat mRNA. Translation from mouse *TrkA* mRNAs with 10b as 5′ terminus most probably starts from exon 12 (at genomic position 87782262) producing TRKAκ protein, similarly to the human orthologue. 3 uORFs up to 114 nt are also present.

We did not observe 5′ exons of rat or mouse *TrkA* alternative to exon 1, 8 or 10 and PCR with primers designed based on sequence similarities with human exons A and D did not give any results in samples from either mouse or rat tissues. *TrkA* genes of mouse and rat therefore do not overlap with either *SH2D2A* or *INSRR* genes, although the latter one is very close to *TrkA* exon 1 in case of both species.

Analysis of* TrkA* transcripts in mouse and rat revealed that both express exons 9a and 10a and therefore, potentially also TRKAκ (Fig. 1e, f). As we did not detect exons 9a or 10a in 5′ RACE experiments using mouse or rat samples, it can be concluded that these exons are produced in these animals only by intron retention and not by alternative transcription initiation mechanism seen in human. In case of rat transcripts with exon 10a, the potential translational start-site is most probably located in the intron between exons 9 and 10 and TRKAξ protein is generated (Fig. 1f). However, mouse and rat mRNAs with exons 9a or 10a have many uORFs, some of which are very large.

Furthermore, exon 9 is a cassette exon in both mouse and rat and exon 11 can be spliced out in mouse. While the splice variants lacking exons 2 and 3 or exons 2…4 have been described in literature, we identified only the latter one [28].

A membrane signal sequence was not detected by Phobius prediction tool for any novel putative TRKA isoforms in rat or mouse with N-termini other than the conventional Met of TRKAI/TRKAII.

### Expression of *TrkA* mRNAs in mouse tissues

The expression of *TrkA* mRNA was studied in a selection of adult neuronal and non-neuronal tissues as well as in developmental samples from mouse brain at gestation day 13 (E13) and at postnatal days 1, 9, 14 and 60 (P1, P9, P14 and P60, respectively; Fig. 3). According to the expression level of exons 12…17, common to all possible splice forms, the samples examined showed relatively similar overall *TrkA* expression levels with the exception of muscle tissue, where *TrkA* mRNA was almost undetectable. The 5′ region of transcripts from heart sample was identified only when the number of PCR cycles was increased (data not shown). The developmental tissues analyzed had a fairly higher expression level of *TrkA*.

In human and rat *TRKA* transcripts, exon 9 was spliced out in non-neuronal tissues and included in neuronal tissues. In mouse, this rule seems not to apply, as *TrkA* mRNAs without exon 9 were observed only in testes as a minor product in addition to the major form with exon 9.

Interestingly, PCR with primers amplifying exons 10a and 9a predominately gave rise to products with exon 9a not with 10a, which is in contrast to our results in human and rat tissues. It can be concluded that the regulation of splicing in the region around exon 9 of the *TrkA* mRNA in mouse is different from that in rat and human.

Furthermore, a splice form lacking exon 11 was detected in mouse testis sample only when exon 9a was included in the transcript.

Analysis of mouse samples provided evidence that alternative transcription initiation and splicing of *TrkA* gene is less complex in mouse than in human. However, a novel splice form, lacking exon 11 and not seen in human, was identified. *TrkA* expression in muscle was almost undetectable in mouse while the overall level of *TRKA* in human muscle tissue was not significantly lower than in other tissues.

### Expression of *TrkA* mRNAs in rat tissues

*TrkA* expression was detected in all rat tissues analyzed (Fig. 3). The overall mRNA levels were higher in developmental brain samples [embryonic day (E) 13, postanatal day (P) 7 and P21], in adult whole brain and sympathetic ganglia and in PC12 cell line, and lowest in heart.

Splice variant lacking exons 2 and 3 was detected in minor quantities in PC12 cells. *TrkA* transcript, where exon 9 has been spliced out, was observed in kidney, testis, muscle, heart—the analyzed non-neuronal tissues, and also in the PC12 cells. In the rest of the samples and also in testis and PC12 cells, the major transcript contained exon 9. The splicing pattern of *TrkA* exon 9 in different rat tissues was similar to human, but different from mouse.

Similarly to human, transcripts with exon 10a were more frequent than transcripts with exon 9a, with the exception of adult whole brain and sympathetic ganglia. In cortex and in P21 brain, neither was detected. Yet in most of the other neuronal tissues and in PC12 cells, both transcript variants were observed. Interestingly, the expression levels of transcripts with exons 10a and 9a were fluctuating during the development: in embryonic brain from gestation day 13, a relatively high level of transcripts with 10a exon was observed as well as relatively low levels of 9a variant. Thereafter, the level of these mRNAs started to decrease, as P7 brain had lower levels of these splice variants and at P21 the signal of *TrkA* mRNAs with exons 10a or 9a was not detected. However, the transcript with exon 9a was observed again in the adult whole brain.

Interestingly, the expression of *TRKA* in human and rat muscle tissues followed a similar pattern while in mouse it was undetectable. Heart displayed contrary expression profiles by having relatively low *TRKA* transcription levels in human and rat and higher levels in mouse.

### Endogenous TRKA protein isoforms in PC12 and SH-SY5Y cells

To examine the expression of *TrkA* on protein level, PC12 cell line from rat was chosen for the experiments as it has been used widely for research on TRKA. Of TRK receptors, it is known to express predominantly TRKA and to be NGF responsive [45]. The SH-SY5Y cell line was added to analyses as it showed the highest levels of *TRKA* mRNA expression among human samples in the RT-PCR studies.

We tested many antibodies and according to results obtained with siRNA treated PC12 cell lysates, the most effective antibody to detect the low levels of endogenous TRKA was an anti-TRKA rabbit polyclonal antibody by Millipore (#06-574, see also “Methods”). The epitope of this antibody localizes to the extracellular region of TRKA protein, consequently it would not be able to recognize several putative isoforms excluding this region. Another promising antibody was an anti-TRK rabbit monoclonal antibody by Cell Signaling (#4609, see also Methods), which recognizes all three TRK receptors and has its epitope around Y785 of TRKA C-terminus. Lastly, we also used an anti-pTRKA antibody from Cell Signaling (#9141, see also “Methods”) that only recognizes phosphorylated TRK proteins. 5 min NGF treatment before the lysis of the cells was used to activate the intrinsic phosphorylation ability of TRKA proteins. Both anti-TRK and anti-pTRKA antibodies are therefore presumably capable of distinguishing all putative TRKA isoforms that contain the kinase domain.

**Fig. 4:**
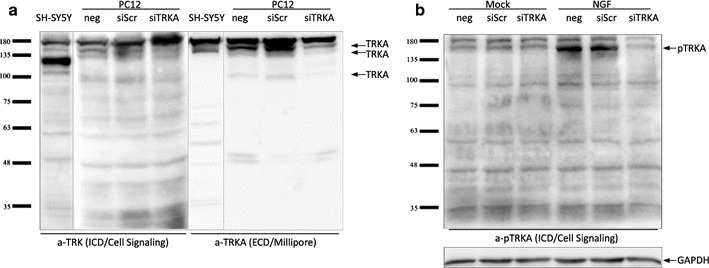
Detection of endogenous TRKA protein in human SH-SY5Y and rat PC12 cell line. **a** 50 µg of lysates of PC12 cells transfected (48 h) with siRNAs and SH-SY5Y cells was separated on SDS-PAGE. Western blot analysis was performed using Cell Signaling anti-TRK [epitope in the intracellular region (ICD) of TRK proteins; #4609] and Millipore anti-TRKA [epitope in the extracellular region (ECD); #06-574] antibodies. **b** PC12 cells were transfected (48 h) with siRNAs and treated for 5 min with NGF. Mock-treated cells were used as control. 60 µg of lysates was subjected to SDS-PAGE and western blotting with antibody against phosphorylated TRKA (Cell Signaling anti-pTRKA; #9141). GAPDH was used to validate the loading efficiency. *siScr* unspecific scrambled siRNA, *siTRKA* rat TRKA specific siRNA, *neg* mock treated cells

In PC12 cells the full-length TRKAI/TRKAII protein of ≈100 kDa and it’s glycosylated forms ≈120 and 140 kDa were detected by both anti-TRK (#4609) and anti-TRKA (#06-574) antibodies (Fig. 4a). Using anti-TRK (#4609) antibody the signal from smaller proteins than 100 kDa was not observed. Similarly, the anti-pTRKA (#9141) antibody detected only the full-length pTRKAI/pTRKAII in its glycosylated form (≈140 kDa; Fig. 4b) which is the type of TRKA expressed on the cell surface and is therefore accessible to extracellular NGF. In our current study we were not able to distinguish with the given antibodies in PC12 cells the predicted novel TRKA isoforms. This could be caused by the poor ability of antibodies to recognize low levels of endogenous TRKA.

In SH-SY5Y cells the signals corresponding to 120 and 140 kDa probably represent the differently glycosylated TRKA proteins. The anti-pTRKA antibody did not detect signals from SH-SY5Y cell lysates even if the cells had been previously treated with NGF (data not shown) suggesting that TRKA receptors are not functional in proliferating SH-SY5Y cells used for analysis.

Interestingly, by all three antibodies used a ~180 kDa signal was detected, which identity remains to be elucidated. If this is indeed TRKA, e.g., a very stable hyper-glycosylated form, it appears to be a type that does not respond to NGF treatment (Fig. 4b).

### TRKAII putative isoforms differ in autocatalysis rate

We were next interested in describing novel putative TRKA isoforms in more detail. Most of the detected human *TRKA* transcripts have out of frame uORFs relative to the ORF that is depicted in Fig. 1c. None of these uORFs reach the 3′ exons of *TRKA*. Due to this, if the longest ORF is not translated, the mRNA would be subjected to NMD, a process which promotes the degradation of mRNAs undergoing premature translation termination. We hypothesized that these transcripts might undergo leaky scanning or reinitiation that would exclude them from NMD as the uORF sequences start with AUG surrounded with weak Kozak sequences [46].

TRKA isoforms with different N-termini can each have several splice forms (except TRKAβ, TRKAθ and TRKAκ); however, the functional implications of the exclusion of six amino acids encoded by exon 9 (resulting in TRKAI, TRKAαI, TRKAγI, etc.) have been described before [23, 24]. Also, transcripts encoding TRKA isoforms other than type I and II showed relatively low level of expression. Accordingly, TRKA isoforms of type I, III, IV, V, VI, VII, VIII and IX were omitted from further analysis for simplicity.

**Fig. 5:**
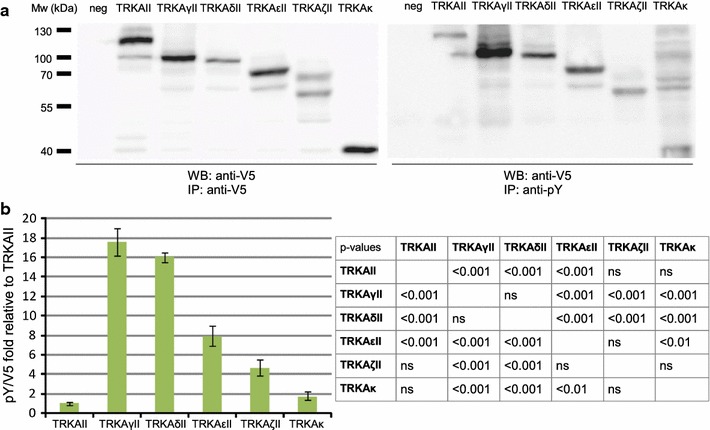
Autophosphorylation potential of a selection of TRKA protein isoforms. HEK293 cells were transfected with constructs encoding different putative TRKA isoforms. Untransfected cells were used as a negative control (neg). Isoforms were precipitated from lysates using rabbit antibody against V5-tag. **a** Equal amounts of immunoprecipitate were analyzed with SDS-PAGE and Western blot using mouse V5-antibody and mouse phosphotyrosine antibody. **b** Immunoblot signals from three independent experiments were quantified and the phosphorylated fractions of proteins were defined as pY/V5 ratios, which were log transformed, mean centered and autoscaled for statistical analysis. Results from Tukey–Kramer multiple comparisons test are compiled into a table. For graphic representation, data is expressed as a fold change relative to the pY/V5 ratio of TRKAII. Back-transformed mean values ± SEM *bars* are shown

To determine whether N-terminal differences of putative TRKA isoforms influence autophosphorylation capacity, a selection of potential TRKA isoforms was cloned and the expression constructs of TRKAII-V5-His, TRKAγII-V5-His, TRKAδII-V5-His, TRKAεII-V5-His, TRKAζII-V5-His and TRKAκ-V5-His were transfected into human embryonic kidney 293 cells (HEK293), followed by lysis and V5-tag-aimed immunoprecipitation to eliminate endogenous phospho-tyrosine (pY)-proteins from Western blot analysis. Total precipitated protein was visualized with an antibody against V5-tag and phosphorylated subportion with anti-pY antibody. All isoforms were expressed efficiently (Fig. 5a, left panel) and displayed catalytic activity (Fig. 5a, right panel). It was repeatedly observed that TRKAκ-V5-His immunoprecipitate contained pY-proteins besides TRKAκ-V5-His. These proteins were of higher molecular weight than the expected ~40 kD TRKAκ-V5-His protein and were not present in precipitates containing other TRKA isoforms. If this was a result of co-precipitation, it can be assumed that these protein interactions are so strong as to withhold the harsh detergent conditions of radioimmunoprecipitation assay (RIPA) lysis buffer. Phosphorylated proteins of unknown origin were not seen with other overexpressed TRKA proteins.

Isoforms seemed to vary in the extent of autophosphorylation. To better characterize this variation, Western blot signals were quantified with densitometric analysis. Data was converted into an anti-pY to anti-V5 signal ratio (pY/V5) for each isoform and presented as a fold change relative to the pY/V5 ratio of TRKAII. Significance of differences of these ratios over three independent experiments was determined with Repeated Measures ANOVA and with a post hoc multiple comparisons test (Tukey–Kramer). The results are shown in Fig. 5b.

When protein expression level is very high, such as in cancer, even those kinase receptors exhibit autocatalysis that under normal physiological conditions are repressed. In the current experiment, the amount of protein monomer was not the sole determinant of autophosphorylation, since for every isoform the strength of V5 signal does not correlate with the strength of pY signal. Most notably, TRKAγII-V5-His and TRKAδII-V5-His were sixteen times more phosphorylated relative to TRKAII-V5-His. pY/V5 ratio difference between TRKAγII-V5-His and TRKAδII-V5-His was not statistically significant, as likely there are no biologically significant differences concerning autophosphorylation rate of these isoforms, since these proteins vary only by the presence or absence of a predicted signal peptide. TRKAεII-V5-His displayed two-fold smaller rate of simultaneous kinase activation as compared to TRKAγII and TRKAδII. This difference might have biological relevance, since TRKAεII protein almost entirely lacks cysteine-flanked leucine-rich motifs that could stimulate dimerisation in TRKAγII and TRKAδII. TRKAκ-V5-His, a protein corresponding to the intracellular domain of TRKAII had an autoactivation capacity comparable to TRKAII, possibly because TRKAκ lacks extracellular domains which could facilitate dimerisation and subsequent autophosphorylation as seen for other putative TRKAII isoforms.

### TRKAζII is a glycoprotein residing in intracellular compartments

**Fig. 6:**
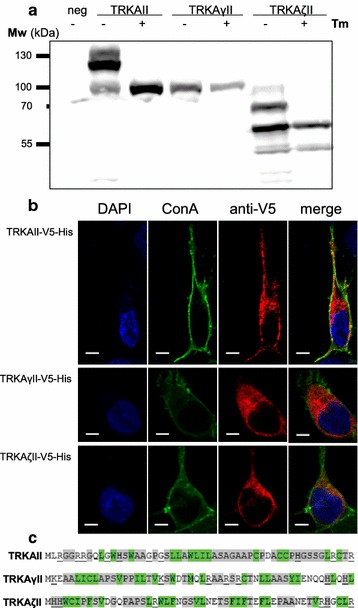
TRKAζII-V5-His is a glycoprotein not targeted to the plasma membrane. HEK293 cells were transfected with plasmids encoding proteins TRKAII-V5-His, TRKAγII-V5-His and TRKAζII-V5-His. The proteins were detected using western blotting and an antibody against the V5-tag. **a** TRKAζII-V5-His is a glycoprotein. Cells were cultured with (+) or without (−) tunicamycin (Tm, 2 µg/ml), the inhibitor of N-linked glycosylation. Untransfected cells were used as a negative control (neg). **b** Intracellular localization pattern of tagged TRKA isoforms detected with immunocytochemical analysis. DNA was stained with DAPI, plasma membrane was visualized with Alexa Fluor 488-conjugated concanavalin A. *Scale* *bar* 5 µm. **c** The first 52 N-terminal amino acid residues of TRKAII, TRKAγII and TRKAζII. Amino acids that are considered to be more hydrophobic are in *green*, less hydrophobic are* grey*, amino acid residues with positive charges are *underlined*

We noticed that TRKAII-V5-His and TRKAζII-V5-His lysates showed signals from larger proteins than can be estimated (Fig. 5a). Based on an analogy to immunoblot pattern observed for TRKAII-V5-His it was assumed that TRKAζII-V5-His could also be glycosylated similarly to TRKAII. To clarify this issue, HEK293 cells were transfected with expression constructs of TRKAII-V5-His, TRKAγII-V5-His and TRKAζII-V5-His, followed by treatment with tunicamycin, an inhibitor of N-linked glycosylation. Lysates were analyzed with an anti-V5 antibody. Tunicamycin inhibition was effective as tunicamycin-treated cells transiently expressing TRKAII-V5-His contained only the unmodified form of the receptor (Fig. 6a). TRKAγII-V5-His was included because it was the only novel putative TRKA isoform for which the Phobius prediction tool estimated a membrane signal sequence and thus it could be directed to the ER-Golgi route where it can be glycosylated. However, since the predicted size of the protein without glycosylation coincided with the Mw of the protein seen on SDS-gel (Fig. 5a), it seemed that the signal sequence of TRKAγII is nonfunctional. In accordance with this finding, tunicamycin did not change the electrophoretic mobility of TRKAγII and the intracellular localization of TRKAγII-V5-His appeared to be cytosolic (Fig. 6b). On the other hand, the disappearance of signal from the protein with bigger Mw in case of TRKAζII-V5-His-transfected and tunicamycin-treated cells is a clear indication that this isoform is modified at least on one of the five consensus N-glycosylation sites (N-X-S/T sequons) it has. This finding seems to place TRKAζII-V5-His to the ER-Golgi route, where N-glycosyltransferases reside. However, as opposed to the full-length TRKAII, TRKAζII did not reach plasma membrane in HEK293 cells transiently transfected with TRKAζII-V5-His expression plasmid (Fig. 6b).

Usually, proteins are targeted to the ER when nascent signal peptide is revealed during the first translational round [47]. Signal peptides are not conserved among secretory and integral proteins, but generally consist of 20–30 residues with positively charged aa residues in the N-terminus followed by a hydrophobic core of at least six residues. However, yet undefined structural features seem to be also important [48]. Phobius prediction tool for signal peptides estimated that TRKAγII could be directed to plasma membrane while TRKAζII cannot. Although TRKAγII has a longer stretch of hydrophobic amino acids in the N-terminus compared to TRKAζII, this hydrophobic core is preceded by a lysine and glutamate, whereas TRKAζII has two histidines in the N-terminus (Fig. 6c). Only about 10 % of histidine residues are in a positively charged state at physiological pH, however, apparently this serves to be a recognizable signal peptide, whereas TRKAγII’s N-terminus does not.

### TRKAκ localizes to the cytoplasm and the nucleus

**Fig. 7:**
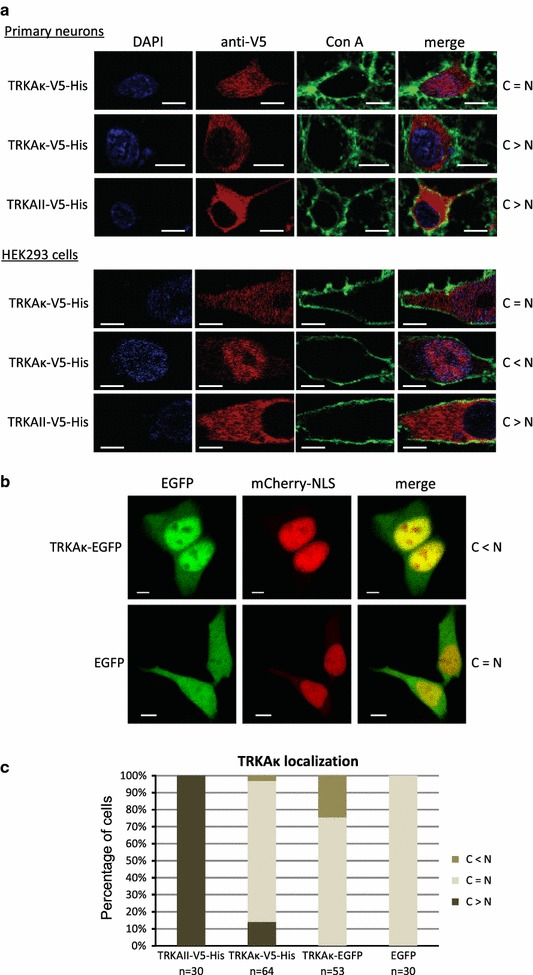
TRKAκ isoform’s localization into the nucleus. **a** immunocytochemical analysis revealing TRKAκ-V5-His and TRKAII-V5-His localization in primary rat neurons and HEK293 cells. DNA was stained with DAPI. To mark glycoproteins embedded in the plasma membrane, Alexa Fluor 488-conjugated concanavalin A (con A) was used, and TRKA-V5-His proteins were visualized with anti-V5-tag antibodies and secondary Alexa Fluor 568-conjugated antibodies. *Scale bar* 5 µm. **b** Nuclear accumulation of EGFP-fused TRKAκ in HEK293 cells. Cells were transfected with constructs encoding TRKAκ-EGFP or EGFP, and mCherry-NLS (the marker for nucleus). Live cells were imaged with a confocal microscope. *Scale bar* 5 µm. **c** HEK293 were fixed and subjected to immunocytochemistry 24 h p.t. of expression constructs encoding TRKAII-V5-His, TRKAκ-V5-His, TRKAκ-EGFP or EGFP. The cells were visualized with confocal microscopy and counted by determining the localization of given protein of interest in n number of cells. The *graph* shows pooled results of two independent experiments. C = N, the signal was observed uniformly in cytosol and nucleus; C > N, the signal was greater in cytosol than in nucleus; C < N, the signal was greater in nucleus than in cytosol

Initial experiments with putative TRKA protein isoforms in HEK293 cells revealed that additionally to cytosolic distribution, TRKAκ-V5-His is also present in the nucleus. To confirm this finding, immunocytochemical analysis was carried out in HEK293 cells and primary rat cortical neurons transfected with the expression construct encoding TRKAκ-V5-His or TRKAII-V5-His as a control. Although TRKAII-V5-His was restrained to the cytoplasm and the plasma membrane, the ability of TRKAκ-V5-His to localize to the nucleus of neuronal cells was confirmed, however, various types of localization were observed (Fig. 7a). Therefore, this effect is not cell-type specific.

Since fixation and permeabilization procedures preceding immunocytochemistry can alter protein localization pattern, the nuclear translocation of TRKAκ was verified in HEK293 cells with live imaging of EGFP-tagged protein by directly observing the fluorescence of EGFP. Moreover, TRKAκ-EGFP has a significantly bigger molecular weight (70 kDa) than TRKAκ-V5-His (42 kDa) and cannot diffuse to the nucleus passively. The control EGFP displayed a uniform localization pattern, while in some cells the signal of TRKAκ-EGFP was clearly accumulated in the cell nucleus (Fig. 7b).

To better describe this phenomenon, we used immunocytochemistry to quantify the intracellular localization of overexpressed TRKAκ-V5-His and TRKAκ-EGFP in HEK293 cells. While the control TRKAII-V5-His was restricted to cytosol and plasma membrane, as expected, TRKAκ with the same tag entered nucleus in more than 80 % of cells and 3 % of cells expressing TRKAκ-V5-His displayed signal that was predominantly in the nucleus (Fig. 7c). On the other hand, EGFP protein was uniformly distributed between cytosol and nucleus in all cells examined. When TRKAκ was linked to EGFP, then the level of signal from cell nucleus exceeded that from the cytosol in 25 % of cells (Fig. 7c). Therefore, our experiments clearly indicate that TRKAκ is able to enter cell nucleus.

There is no conventional nuclear localization signal (NLS) present in TRKA. Nevertheless, in the proximity of N-terminus of TRKAκ a signal sequence SPT that has been shown to mediate the recognition by Importin-7 (Imp7) was identified. There is numerous evidence of Imp7 translocating a variety of proteins such as Smads, extracellular signal-regulated kinase 2 (Erk2) and early growth response protein 1 and glucocorticoid receptor (Egr-1) to the nucleus [49–52]. A hypothesis of the translocation of TRKAκ to the nucleus by Imp7 via the SPT sequence was proposed. Cellular localization of TRKAκ-SPT-EGFP (lacking the putative signal sequence) bared no significant difference with TRKAκ-EGFP (data not shown). Therefore, it can be concluded that the sequence eliminated was not responsible for the accumulation of the TRKA intracellular fragment to the nucleus and Imp7 was most probably not involved in the transportation of TRKAκ to the nucleus.

## Discussion

### *TRKA* gene organization and expression in human, mouse and rat

As more and more mRNA sequence information becomes available, it is becoming evident that overlapping genes are more common than previously thought [53, 54]. Even so, gene trios seem to be relatively rare [44]. In this study we describe *TRKA* gene, which in human is overlapped by two genes of the opposite strand—*INSRR* and *SH2D2A*. More specifically, these genes are overlapped by novel *TRKA 5′* exons A…D with the transcription-initiation site in exon A. Despite our efforts, we were unable to identify these exons in mouse or rat. As the transcription-initiation sites of human *TRKA* exon A and *SH2D2A* are less than 1 kb apart, it can be speculated that the unidirectional promoter of *SH2D2A* has become bidirectional at one point in the evolutionary history of human, therefore giving rise to this novel 5′ terminus of *TRKA*. There is probably a complex interaction between the expression of these three genes, as they might influence each other in synergistic or competitive way during and after transcription. For example, the close proximity of *INSRR* and *TRKA* promoters might render the genomic region accessible to transcriptional machinery simultaneously for both genes. In accordance with this, it has been shown that *TRKA* and *INSRR* expression patterns are highly similar [55]. On the other hand, *TRKA* exon D overlaps an exon of *INSRR* gene by more than 80 bp and this might result in partly double-stranded mRNAs and gene silencing as the respective regions in mRNA transcripts are complementary.

It should be noted that while we did not identify novel 5′ exons of rodent *TrkA*, no previous 5′ RACE analyses of rat *TrkA* were recorded in literature to our knowledge. We identified numerous additional transcription-initiation sites and alternative splicing patterns of human *TRKA* which indicate the possible existence of many protein isoforms. Overall—all of the tissues under study expressed many types of 5′ *TRKA* exons. The translatability of many of these proteins could be debated as most of these mRNAs contain uORFs. However, according to bioinformatics studies, approximately half of mammalian protein-coding transcripts contain uORFs [56]. It has been shown that even when a strong Kozak sequence surrounds the AUG in uORF or when up to four uORFs precede the main ORF, nearly 10 % of ribosomes are capable of leaky scanning or reinitiation up to five times [46]. The alternative transcripts carrying several uORFs could also use these sequences as a regulatory element for adjusting the specific protein levels to match the necessities of the cell. Also, some of the uORFs may be inaccessible for ribosomes due to RNA secondary structure. It remains to be determined which of these novel transcripts represent functional *TRKA* mRNAs and which can be considered as transcriptional noise.

### Characteristics of putative novel TRKA protein isoforms

In TRK proteins, a kinase monomer is kept in an inactive conformation through autoinhibition mechanism (the projection of one tyrosine residue into the active site). However, since kinase domain structure allows the movement of its parts to some degree, every monomer is occasionally freed from inhibited state and is able to phosphorylate in trans [57]. This spontaneous activity is deterred by the properties of the extracellular domain, such as the presence of glycan side chains [21, 22]. In accordance to this, the autocatalysis rates of potential TRKA isoforms tested in this study were very different with the highest levels seen for TRKAγII, TRKAδII and TRKAεII. All these isoforms contain part of the extracellular domain of the prototypic TRKAII and are not glycosylated, as indicated by protein mobility pattern on SDS-PAGE and the lack of functional membrane signal sequences which renders them inaccessible to N-glycosyltransferases that are located in the Golgi apparatus. Phosphorylation of TRKAγII and TRKAδII could also be enhanced by the presence of unique amino acid residue stretch encoded by exons A and D.

We identified a putative TRKA isoform TRKAζII which was glycosylated in the ER-Golgi route and must therefore possess a functional membrane-signal sequence. However, when overexpressed, this protein was not transported to the plasma membrane, but got stalled intracellularly. Similarly, TRKAIII is also known to be retained intracellularly with a highly autoactivated TRKAIII-pool residing in the ER/Golgi intermediate compartment [27]. It has been suggested that TRKAIII is not directed to the plasma membrane, because it lacks a site for the addition of a glycan moiety acting as a signal for further translocation. On the basis of current study, TRKAζII-V5-His seems to be confined to intracellular compartments similarly to TRKAIII. TRKAζII protein contains the juxtamembrane region that is omitted in TRKAIII but just as TRKAIII it does not have the first Ig-like domain. Thus, the first Ig-like domain could serve an important regulatory function in the trafficking of TRKA protein.

Among several alternative TRKA variants, an interesting putative isoform, termed TRKAκ, emerged from our study. The protein constitutes primarily of the receptor’s tyrosine kinase domain. There are several reasons why this variant drew our attention. First, it is noteworthy that while the alternative splicing pattern observed in rat and mouse bares little similarities to the complexity seen in humans, the expression of transcripts with exons 9a and 10a that contribute to TRKAκ-encoding mRNAs was detected in all species examined. Second, these mRNAs have a distinct expression pattern in different tissues, leaving room for further speculation about the possible importance of this isoform in diverse cellular frameworks. The rationale for further investigations of this protein includes also the possibility that proteolysis may generate a TRKAκ-like fragment, as under excitotoxic conditions or in response to amyloid-β peptide the highly similar TRKB receptor has been recently shown to be subjected to proteolytic cleavage, resulting in a kinase-domain containing soluble protein fragment [58–60]. This type of process has also been detected for another receptor tyrosine kinase called erythroblastic leukemia viral oncogene homolog-4 (ErbB-4). The ectodomain of ErbB-4 is cut by metalloprotease and the intracellular domain that is cleaved by γ-secretase is thereafter translocated to nucleus [61]. Cleavage of the TRKA extracellular domain producing an ectodomain fragment and membrane-bound truncated TRKA fragments with intracellular kinase domain has been demonstrated, but no soluble fraction of TRKA kinase domain has yet been discovered [62].

The most intriguing property of TRKAκ is undoubtedly its intracellular localization which varies from cytosolic to nuclear. Under physiological conditions passive flux through nuclear pore complex is very restricted for molecules bigger than 30 kDa [63]. Thus, a TRKAκ-like protein (35 kDa) especially when it’s EGFP-tagged (Σ ≈ 70 kDa) would only gain access to the nucleus with the aid of active transport or facilitated diffusion. Appearance of distribution patterns where TRKAκ-V5-His or TRKAκ-EGFP signal was more strongly detected in the cytosol or the nucleus could suggest that the movement of TRKAκ to the nucleus is regulated, since active transport of TRKAκ might be dependent on the availability of adapter proteins that are only expressed in a certain time frame during cell-cycle or under certain cellular physiological states. Alternatively, TRKAκ could shift towards passive nuclear accumulation when high-affinity nuclear anchoring proteins in the nucleus do not allow its export and/or impede its free diffusion.

Previously, by using the method of immunocytochemistry, TRKA has been detected in the nuclei of various cells, such as melanocytic tumors [64], ovarian carcinoma [65], human glioma cell line U251 [66], rat pheochromocytoma cell line PC12 and cultured hepatic stellate cells [67]. In liver cells TRKA nuclear immunoreactivity was observed when antibody against C-terminus was used, whereas antibody against N-terminus did not reveal TRKA. Overall, it is not clear whether nuclear TRKA seen in those studies represents the whole receptor, its proteolytic fragment, an alternative isoform or non-specific staining. Our current study implies that neither HEK293 cells nor rat primary neurons contain transporting machinery necessary for whole receptor translocation into the nucleus, although this could possibly be otherwise in different cell types. However, our results established that TRKAκ tagged to V5-His or EGFP is definitely present in the nucleus whether it is encoded by specific mRNAs or proteolytically generated from the full-length receptor.

As compared to many other TRKA isoforms, TRKAκ displayed relatively low autocatalysis rate which is particularly interesting in the light of the finding that TRKAκ was bound to other pY-proteins, while for more highly phosphorylated isoforms of TRKA it was not possible to detect interactions of this intensity. Presumably only the small p-TRKAκ fraction was capable of interacting with these proteins, since they were tyrosine-phosphorylated and it can be assumed that this modification was mediated by the kinase activity of p-TRKAκ. Alternatively, the situation may be reversed as some of these proteins may be tyrosine kinases phosphorylating TRKAκ. However, given that this was the only TRKA isoform displaying nuclear localization, it can be suggested that there are high-affinity substrates for TRKA that may reside inside the nucleus.

Identical protein to TRKAκ was characterized by Coulier and coworkers when different deletion-mutants of TRKA were assessed on the ability to transform NIH 3T3 cells [68]. They found that this protein is a functional kinase, but has no transforming ability just as the full-length receptor. Interestingly, in melanomas, nuclear expression of phosphorylated TRKA was more pronounced in primary tumors relative to metastases [64]; however, in ovarian carcinoma nuclear expression was not more characteristic to any stage of cancer progression [65]. Thus, it is unclear whether TRKA nuclear activity contributes to malignant phenotype or aids to maintain a stable state.

The novel cytoplasmic and nuclear isoforms of TRKA cannot be activated by NGF or NT-3 because neurotrophins are directed to membrane-bound ER-lumen and to the vesicles of the Golgi complex already during their synthesis. Therefore, their interaction with those TRKA isoforms is sterically impossible. However, these TRKA proteins can undergo spontaneous autoactivation or, alternatively, there might be some other activating factors within the cell which are yet unidentified.

## Conclusions

TRK receptors have crucial roles in processes with various outcomes such as proliferation, survival and differentiation. Thus, the activity of these receptors has to be regulated for the correct cellular fate. Control at mRNA level through alternative splicing and several alternative transcription initiation sites provides mechanisms to diversify the pool of TRK proteins with different properties.

In this study, *TRKA* transcripts were studied in silico, by 5′ RACE and by semiquantitative analyses. The expression patterns of alternative *TRKA* transcripts were analyzed in different human brain regions and peripheral human, mouse and rat tissues. Many novel alternative transcript variants were detected in human tissues and the presence of a large number of TRKA protein isoforms was predicted that differ in N-termini and protein sequences of the extracellular domain encoded by alternatively spliced exons. In rat and mouse tissues the splicing observed was less intricate. Our experiments showed that soluble TRKA isoforms, which contain parts of unglycosylated extracellular domain, are highly autocatalytic in comparison to plasma membrane-embedded glycosylated TRKAII receptor. One of the putative isoforms, TRKAζII, is a glycoprotein residing in intracellular compartments similarly to isoform TRKAIII. Therefore, it can be inferred that the first Ig-like domain in TRKAI/II that is missing in both TRKAIII and TRKAζII, is necessary for the translocation of receptor to the plasma membrane. One of the putative isoforms that is composed mainly of the kinase domain, named TRKAκ, displayed a relatively low level of autocatalysis rate. Interestingly, TRKAκ was detected in the nucleus and cytoplasm of transiently transfected fixed as well as live cells.

These findings lay ground to future studies in the field of alternative TRKA isoforms, as there seems to be an immensely larger variability among TRKA proteins with different properties than is presently known.

## Methods

All experiments with human postmortem tissues were approved by the ethics committee of medical studies at National Institute for Health Development of Estonia (Permit Number: 402). The protocols involving animals were approved by the ethics committee of animal experiments at Ministry of Agriculture of Estonia (Permit Number: 45). Human RNAs used in this study were acquired from BD Biosciences (thymus, muscle, heart, prostate, testis, pancreas, kidney and colon samples), from BioChain Inc. (human spleen and fetal tissues), or were extracted from frozen adult human postmortem brain regions obtained from North Estonian Regional Hospital, Tallinn. Rat tissues were obtained from Sprague–Dawley rats and mouse tissues from NMRI mice housed under a 12 h light/dark cycle in local animal facility with *ad libitum* access to water and food.

### RT-PCR and 5′ RACE

In silico analysis of the *TRKA* gene structure and transcripts, reverse transcription and PCR methodology have been described before [69]. 5′ RACE experiments were conveyed with GeneRacer Kit (Invitrogen) according to the manufacturer’s protocol (Invitrogen). For PCR, HotFire polymerase from Solis Biodyne was used. All primers used in this study are listed in Additional file 2, *TRKA* ESTs identified with sequencing have been submitted and the corresponding GenBank accession numbers can be found in Additional file 3.

### Generation of expression constructs

To generate the V5-His-tagged TRKAII isoform, PCR was conducted to amplify the coding region from human frontal cerebral cortex cDNA and cloned into pcDNA3.1 (Invitrogen). Sequences encoding N-terminal parts of different TRKA isoforms were amplified from human frontal lobe or muscle cDNA and ligated with plasmid pTZ57R/T of InsTAclone™ PCR Cloning Kit (Fermentas). Verified sequences were subcloned into the pcDNA3.1/TRKAII-V5-His vector using the following restriction enzymes: *Hin*dIII and *Nar*I (for pcDNA3.1/TRKAγII-V5-His), *Xba*I and *Eco*47III (for pcDNA3.1/TRKAδII-V5-His), *Hin*dIII and *Bal*I (for pcDNA3.1/TRKAεII-V5-His), *Hin*dIII and *Pag*I (for pcDNA3.1/TRKAζII-V5-His), *Xba*I and *Nco*I (for pcDNA3.1/TRKAκ-V5-His).

EGFP-tagged TRKAκ was generated by excising the V5-His tag-coding sequence from the pcDNA3.1/TRKAκ-V5-His plasmid and substituting it with EGFP-coding sequence from pEGFP-N2 (Clontech). For this, Cfr42I (in case of pcDNA3.1/TRKAκ-V5-His) and *Not*I (for pEGFP-N2) restriction enzymes were used, followed by DNA-blunting with T4 DNA polymerase in the presence of dNTPs and a final restriction with *Bam*HI enzyme. Restriction products of interest were ligated. All restriction enzymes were purchased from Fermentas and all DNA constructs were verified by sequencing.

### Cell culture and transfection

HEK293 cells were grown in Minimum Essential Medium (MEM) with Earle’s salts and l-Glutamine containing 10 % fetal bovine serum (FBS) and 1 % penicillin/streptomycin. LipoD293™ DNA In Vitro Transfection Reagent (SignaGen) was used in HEK293 cell transfections. PC12 cells were maintained in Dulbecco’s Modified Eagle’s Medium (DMEM) containing 6 % FBS, 6 % horse serum (HS) and 1 % penicillin/streptomycin. SH-SY5Y cells were grown in DMEM/Ham’s F12 in 1:1 ratio, containing 10 % FBS and 1 % penicillin/streptomycin. For NGF treatments, growth medium with 50 ng/ml of NGF (PeproTech) was added 5 min prior to harvesting the cell culture. All growth media components were purchased from PAA Laboratories GmbH.

Cerebral cortex was dissected from Sprague–Dawley rat embryos at embryonic day 21. Cells were dissociated with 0.25 % trypsin (Invitrogen), followed by treatment with 0.05 % DNase I (Roche). Cells were grown on poly-l-lysine-coated cover slips in Neurobasal A medium (Invitrogen) with B27 supplement (Invitrogen), 1 % penicillin/streptomycin, and 1 mM l-glutamine (PAA Laboratories GmbH). Mitotic inhibitor 5-fluoro-2′-deoxyuridine (Sigma) was added to the medium (10 μm) at 2 days in vitro (DIV). Primary neurons were transfected at 7 DIV using Lipofectamine 2000 transfection reagent (Invitrogen) as advised by the manufacturer.

The Silencer Select small interfering RNA (siRNA), with nucleotide sequence GUACUUCAGUGAUACCUGUtt, targeting rat *TrkA* and negative unspecific siRNA (#1) were from Ambion (Life Technologies). SiRNAs were transfected to PC12 cells with Lipofectamine RNAiMax Transfection Reagent (Invitrogen) with final 10 nM siRNA concentration, according to the manufacturer’s instructions. Cells were harvested 24–48 h after transfection.

### Western blotting

Western blotting has been described previously [70]. Antibodies used included: rabbit anti-TRKA (#06-574; 1:1000) and mouse anti-pY (#05-1050; 1:2000) from Millipore; rabbit anti-TRK (#4609; 1:500) and rabbit anti-phospho-TRKA (#9141; 1:1000) from Cell Signaling; mouse anti-V5 (#R960-25; 1:5000) from Invitrogen; mouse anti-GAPDH (#G8795; 1:5000) from Sigma-Aldrich.

N-linked glycosylation was inhibited from 6 h after transfection by adding tunicamycin (2 µg/ml; AppliChem) and cells were lysed 9 h later in RIPA buffer.

Immunoprecipitation, immunofluorescence and confocal microscopy were done as described previously [70].

### Live imaging

All live imaging experiments were done at 37 °C, in a chamber supplied with 5 % CO_2_. Zeiss LSM 5 DUO confocal laser scanning microscope with Zeiss confocal scan software was used for imaging. Coverslips containing cultured HEK293 cells were transferred into a metal chamber. For all experiments a 63× glycine immersion fluorescence objective (LSI Plan-Neofluar 63×/1,3 Imm Korr DIC M27) was used.

## Additional files

10.1186/s12868-015-0215-x Sequences of putative TRKA protein isoforms. Red indicates sequences that are not found in TRKAII isoform. (A) Isoforms with different N-termini compared to TRKAII. (B) Exclusion of parts of the extracellular domain as exemplified in the case of isoforms with the conventional N-terminus.
10.1186/s12868-015-0215-x Primers and cycling conditions used in this study.
10.1186/s12868-015-0215-x GenBank accession numbers of ESTs identified in this study.

## Abbreviations

BLAST: basic local alignment search tool
BDNF: brain-derived neurotrophic factor
CIPA: congenital insensitivity to pain with anhidrosis
DIV: days in vitro
DMEM: Dulbecco’s Modified Eagle’s Medium
Egr-1: early growth response protein 1 and glucocorticoid receptor
ER: endoplasmatic reticulum
ErbB-4: erythroblastic leukemia viral oncogene homolog-4
EST: expressed sequence tag
Erk2: extracellular signal-regulated kinase 2
FBS: fetal bovine serum
GPCRs: G protein–coupled receptors
HS: horse serum
HEK293: human embryonic kidney cells
Ig-like: immunoglobulin-like
Imp7: Importin-7
INSRR: insulin receptor-related protein
MEM: minimum essential medium
NGF: nerve growth factor
NTRK1: neurotrophic tyrosine kinase, receptor, type 1
NT-4: neurotrophin-4
NMD: nonsense mediated decay
NLS: nuclear localization signal
PI3 K: phosphatidyl-inositol 3 kinase
pY: phospho-tyrosine
PLC-γ1: phospholipase C-γ1
RIPA: radioimmunoprecipitation assay
5′ RACE: rapid amplification of 5′ complementary DNA ends
Ras-MAPK: rat sarcoma/mitogen-activated protein kinase
RT-PCR: reverse transcription polymerase chain reaction
SH2D2A: sarcoma protein homology 2 domain protein 2A
TRKA: tropomyosin-related kinase A
uORF: upstream ORF
