# Supplementary material for: Novel transcripts reveal a complex structure of the human TRKA gene and imply the presence of multiple protein isoforms
Source: BMC Neurosci. 2015 Nov 18;16:78. doi: 10.1186/s12868-015-0215-x (PMC4652384; doi:10.1186/s12868-015-0215-x)
Supplement: Supplementary file 1 — 10.1186/s12868-015-0215-x Sequences of putative TRKA protein isoforms. Red indicates sequences that are not found in TRKAII isoform. (A) Isoforms with different N-termini compared to TRKAII. (B) Exclusion of parts of the extracellular domain as exemplified in the case of isoforms with the conventional N-terminus. [file 12868_2015_215_MOESM1_ESM.pdf]

A

```
TrkAII    1     --------------------------MLRGGRRGQLGWHSWAAGPGSLLAWLILASAGAAPCPDACCPHGSSGLRCTRDG  54
TrkAαII   1     MSGEAWQLQLGAHRRLPRLSEAGAAAMLRGGRRGQLGWHSWAAGPGSLLAWLILASAGAAPCPDACCPHGSSGLRCTRDG  80
TrkAβ           --------------------------------------------------------------------------------
TrkAγII   1     --------------------------------------------------------MKEAALICLAPSVPPILTVKSWDT  24
TrkAδII         --------------------------------------------------------------------------------
TrkAεII         --------------------------------------------------------------------------------
TrkAζII         --------------------------------------------------------------------------------
TrkAηII         --------------------------------------------------------------------------------
TrkAθ           --------------------------------------------------------------------------------
TrkAκ           --------------------------------------------------------------------------------

TrkAII    55    ALDSLHHLPGAENLTELYIENQQHLQHLELRDLRGLGELRNLTIVKSGLRFVAPDAFHFTPRLSRLNLSFNALESLSWKT  134
TrkAαII   81    ALDSLHHLPGAENLTELYIENQQHLQHLELRDLRGLGELRNLTIVKSGLRFVAPDAFHFTPRLSRLNLSFNALESLSWKT  160
TrkAβ           --------------------------------------------------------------------------------
TrkAγII   25    MQLRAARSRCTNLLAASYIENQQHLQHLELRDLRGLGELRNLTIVKSGLRFVAPDAFHFTPRLSRLNLSFNALESLSWKT  104
TrkAδII   1     MQLRAARSRCTNLLAASYIENQQHLQHLELRDLRGLGELRNLTIVKSGLRFVAPDAFHFTPRLSRLNLSFNALESLSWKT  80
TrkAεII         --------------------------------------------------------------------------------
TrkAζII         --------------------------------------------------------------------------------
TrkAηII         --------------------------------------------------------------------------------
TrkAθ           --------------------------------------------------------------------------------
TrkAκ           --------------------------------------------------------------------------------

TrkAII    135   VQGLSLQELVLSGNPLHCSCALRWLQRWEEEGLGGVPEQKLQCHGQGPLAHMPNASCGVPTLKVQVPNASVDVGDDVLLR  214
TrkAαII   161   VQGLSLQELVLSGNPLHCSCALRWLQRWEEEGLGGVPEQKLQCHGQGPLAHMPNASCGVPTLKVQVPNASVDVGDDVLLR  240
TrkAβ           --------------------------------------------------------------------------------
TrkAγII   105   VQGLSLQELVLSGNPLHCSCALRWLQRWEEEGLGGVPEQKLQCHGQGPLAHMPNASCGVPTLKVQVPNASVDVGDDVLLR  184
TrkAδII   81    VQGLSLQELVLSGNPLHCSCALRWLQRWEEEGLGGVPEQKLQCHGQGPLAHMPNASCGVPTLKVQVPNASVDVGDDVLLR  160
TrkAεII   1     ---------------------------------------------------MPNASCGVPTLKVQVPNASVDVGDDVLLR  29
TrkAζII         --------------------------------------------------------------------------------
TrkAηII         --------------------------------------------------------------------------------
TrkAθ           --------------------------------------------------------------------------------
TrkAκ           --------------------------------------------------------------------------------

TrkAII    215   CQVEGRGLEQAGWILTELEQSATVMKSGGLPSLGLTLANVTSDLNRKNVTCWAENDVGRAEVSVQVNVSFPASVQLHTAV  294
TrkAαII   241   CQVEGRGLEQAGWILTELEQSATVMKSGGLPSLGLTLANVTSDLNRKNVTCWAENDVGRAEVSVQVNVSFPASVQLHTAV  320
TrkAβ           --------------------------------------------------------------------------------
TrkAγII   185   CQVEGRGLEQAGWILTELEQSATVMKSGGLPSLGLTLANVTSDLNRKNVTCWAENDVGRAEVSVQVNVSFPASVQLHTAV  264
TrkAδII   161   CQVEGRGLEQAGWILTELEQSATVMKSGGLPSLGLTLANVTSDLNRKNVTCWAENDVGRAEVSVQVNVSFPASVQLHTAV  240
TrkAεII   30    CQVEGRGLEQAGWILTELEQSATVMKSGGLPSLGLTLANVTSDLNRKNVTCWAENDVGRAEVSVQVNVSFPASVQLHTAV  109
TrkAζII         --------------------------------------------------------------------------------
TrkAηII         --------------------------------------------------------------------------------
TrkAθ           --------------------------------------------------------------------------------
TrkAκ           --------------------------------------------------------------------------------

TrkAII    295   EMHHWCIPFSVDGQPAPSLRWLFNGSVLNETSFIFTEFLEPAANETVRHGCLRLNQPTHVNNGNYTLLAANPFGQASASI  374
TrkAαII   321   EMHHWCIPFSVDGQPAPSLRWLFNGSVLNETSFIFTEFLEPAANETVRHGCLRLNQPTHVNNGNYTLLAANPFGQASASI  400
TrkAβ           -----------------------------------------------------------------------MPAAPTAPR   1
TrkAγII   265   EMHHWCIPFSVDGQPAPSLRWLFNGSVLNETSFIFTEFLEPAANETVRHGCLRLNQPTHVNNGNYTLLAANPFGQASASI  344
TrkAδII   241   EMHHWCIPFSVDGQPAPSLRWLFNGSVLNETSFIFTEFLEPAANETVRHGCLRLNQPTHVNNGNYTLLAANPFGQASASI  320
TrkAεII   110   EMHHWCIPFSVDGQPAPSLRWLFNGSVLNETSFIFTEFLEPAANETVRHGCLRLNQPTHVNNGNYTLLAANPFGQASASI  189
TrkAζII   1     -MHHWCIPFSVDGQPAPSLRWLFNGSVLNETSFIFTEFLEPAANETVRHGCLRLNQPTHVNNGNYTLLAANPFGQASASI  79
TrkAηII         --------------------------------------------------------------------------------
TrkAθ           --------------------------------------------------------------------------------
TrkAκ           --------------------------------------------------------------------------------

TrkAII    375   MAAFMDNPFEFNPEDPIPVSFSPVDTNSTSGDPVEKKDETPFGVSVAVGLAVFACLFLSTLLLVLNKCGRRNKFGINRPA  454
TrkAαII   401   MAAFMDNPFEFNPEDPIPVSFSPVDTNSTSGDPVEKKDETPFGVSVAVGLAVFACLFLSTLLLVLNKCGRRNKFGINRPA  480
TrkAβ     10    DCDAPGMGPWIASTTCPAQRTRLSYTNSTSGDPVEKKDETPFGVSVAVGLAVFACLFLSTLLLVLNKCGRRNKFGINRPA  89
TrkAγII   345   MAAFMDNPFEFNPEDPIPVSFSPVDTNSTSGDPVEKKDETPFGVSVAVGLAVFACLFLSTLLLVLNKCGRRNKFGINRPA  424
TrkAδII   321   MAAFMDNPFEFNPEDPIPVSFSPVDTNSTSGDPVEKKDETPFGVSVAVGLAVFACLFLSTLLLVLNKCGRRNKFGINRPA  400
TrkAεII   190   MAAFMDNPFEFNPEDPIPVSFSPVDTNSTSGDPVEKKDETPFGVSVAVGLAVFACLFLSTLLLVLNKCGRRNKFGINRPA  269
TrkAζII   80    MAAFMDNPFEFNPEDPIPVSFSPVDTNSTSGDPVEKKDETPFGVSVAVGLAVFACLFLSTLLLVLNKCGRRNKFGINRPA  159
TrkAηII   1     MAAFMDNPFEFNPEDPIPVSFSPVDTNSTSGDPVEKKDETPFGVSVAVGLAVFACLFLSTLLLVLNKCGRRNKFGINRPA  74
TrkAθ     1     ----------------------------------------MRQVSVAVGLAVFACLFLSTLLLVLNKCGRRNKFGINRPA  40
TrkAκ           --------------------------------------------------------------------------------

TrkAII    455   VLAPEDGLAMSLHFMTLGGSSLSPTEGKGSGLQGHIIENPQYFSDACVHHIKRRDIVLKWELGEGAFGKVFLAECHNLLP  534
TrkAαII   481   VLAPEDGLAMSLHFMTLGGSSLSPTEGKGSGLQGHIIENPQYFSDACVHHIKRRDIVLKWELGEGAFGKVFLAECHNLLP  560
TrkAβ     90    VLAPEDGLAMSLHFMTLGGSSLSPTEGKGSGLQGHIIENPQYFSDACVHHIKRRDIVLKWELGEGAFGKVFLAECHNLLP  169
TrkAγII   425   VLAPEDGLAMSLHFMTLGGSSLSPTEGKGSGLQGHIIENPQYFSDACVHHIKRRDIVLKWELGEGAFGKVFLAECHNLLP  504
TrkAδII   401   VLAPEDGLAMSLHFMTLGGSSLSPTEGKGSGLQGHIIENPQYFSDACVHHIKRRDIVLKWELGEGAFGKVFLAECHNLLP  480
TrkAεII   270   VLAPEDGLAMSLHFMTLGGSSLSPTEGKGSGLQGHIIENPQYFSDACVHHIKRRDIVLKWELGEGAFGKVFLAECHNLLP  349
TrkAζII   160   VLAPEDGLAMSLHFMTLGGSSLSPTEGKGSGLQGHIIENPQYFSDACVHHIKRRDIVLKWELGEGAFGKVFLAECHNLLP  239
TrkAηII   75    VLAPEDGLAMSLHFMTLGGSSLSPTEGKGSGLQGHIIENPQYFSDACVHHIKRRDIVLKWELGEGAFGKVFLAECHNLLP  154
TrkAθ     41    VLAPEDGLAMSLHFMTLGGSSLSPTEGKGSGLQGHIIENPQYFSDACVHHIKRRDIVLKWELGEGAFGKVFLAECHNLLP  120
TrkAκ     1     ---------MSLHFMTLGGSSLSPTEGKGSGLQGHIIENPQYFSDACVHHIKRRDIVLKWELGEGAFGKVFLAECHNLLP  71
```

```
TrkAII    535  EQDKMLVAVKALKEASESARQDFQREAELLTMLQHQHIVRFFGVCTEGRPLLMVFEYMRHGDLNRFLRSHGPDAKLLAGG  614
TrkAαII   561  EQDKMLVAVKALKEASESARQDFQREAELLTMLQHQHIVRFFGVCTEGRPLLMVFEYMRHGDLNRFLRSHGPDAKLLAGG  640
TrkAβ     170  EQDKMLVAVKALKEASESARQDFQREAELLTMLQHQHIVRFFGVCTEGRPLLMVFEYMRHGDLNRFLRSHGPDAKLLAGG  249
TrkAγII   505  EQDKMLVAVKALKEASESARQDFQREAELLTMLQHQHIVRFFGVCTEGRPLLMVFEYMRHGDLNRFLRSHGPDAKLLAGG  584
TrkAδII   481  EQDKMLVAVKALKEASESARQDFQREAELLTMLQHQHIVRFFGVCTEGRPLLMVFEYMRHGDLNRFLRSHGPDAKLLAGG  560
TrkAεII   350  EQDKMLVAVKALKEASESARQDFQREAELLTMLQHQHIVRFFGVCTEGRPLLMVFEYMRHGDLNRFLRSHGPDAKLLAGG  429
TrkAζII   240  EQDKMLVAVKALKEASESARQDFQREAELLTMLQHQHIVRFFGVCTEGRPLLMVFEYMRHGDLNRFLRSHGPDAKLLAGG  319
TrkAηII   155  EQDKMLVAVKALKEASESARQDFQREAELLTMLQHQHIVRFFGVCTEGRPLLMVFEYMRHGDLNRFLRSHGPDAKLLAGG  234
TrkAθ     121  EQDKMLVAVKALKEASESARQDFQREAELLTMLQHQHIVRFFGVCTEGRPLLMVFEYMRHGDLNRFLRSHGPDAKLLAGG  200
TrkAκ     72   EQDKMLVAVKALKEASESARQDFQREAELLTMLQHQHIVRFFGVCTEGRPLLMVFEYMRHGDLNRFLRSHGPDAKLLAGG  151

TrkAII    615  EDVAPGPLGLGQLLAVASQVAAGMVYLAGLHFVHRDLATRNCLVGQGLVVKIGDFGMSRDIYSTDYYRVGGRTMLPIRWM  694
TrkAαII   641  EDVAPGPLGLGQLLAVASQVAAGMVYLAGLHFVHRDLATRNCLVGQGLVVKIGDFGMSRDIYSTDYYRVGGRTMLPIRWM  720
TrkAβ     250  EDVAPGPLGLGQLLAVASQVAAGMVYLAGLHFVHRDLATRNCLVGQGLVVKIGDFGMSRDIYSTDYYRVGGRTMLPIRWM  329
TrkAγII   585  EDVAPGPLGLGQLLAVASQVAAGMVYLAGLHFVHRDLATRNCLVGQGLVVKIGDFGMSRDIYSTDYYRVGGRTMLPIRWM  664
TrkAδII   561  EDVAPGPLGLGQLLAVASQVAAGMVYLAGLHFVHRDLATRNCLVGQGLVVKIGDFGMSRDIYSTDYYRVGGRTMLPIRWM  640
TrkAεII   430  EDVAPGPLGLGQLLAVASQVAAGMVYLAGLHFVHRDLATRNCLVGQGLVVKIGDFGMSRDIYSTDYYRVGGRTMLPIRWM  509
TrkAζII   320  EDVAPGPLGLGQLLAVASQVAAGMVYLAGLHFVHRDLATRNCLVGQGLVVKIGDFGMSRDIYSTDYYRVGGRTMLPIRWM  399
TrkAηII   235  EDVAPGPLGLGQLLAVASQVAAGMVYLAGLHFVHRDLATRNCLVGQGLVVKIGDFGMSRDIYSTDYYRVGGRTMLPIRWM  314
TrkAθ     201  EDVAPGPLGLGQLLAVASQVAAGMVYLAGLHFVHRDLATRNCLVGQGLVVKIGDFGMSRDIYSTDYYRVGGRTMLPIRWM  280
TrkAκ     152  EDVAPGPLGLGQLLAVASQVAAGMVYLAGLHFVHRDLATRNCLVGQGLVVKIGDFGMSRDIYSTDYYRVGGRTMLPIRWM  231

TrkAII    695  PPESILYRKFTTESDVWSFGVVLWEIFTYGKQPWYQLSNTEAIDCITQGRELERPRACPPEVYAIMRGCWQREPQQRHSI  774
TrkAαII   721  PPESILYRKFTTESDVWSFGVVLWEIFTYGKQPWYQLSNTEAIDCITQGRELERPRACPPEVYAIMRGCWQREPQQRHSI  800
TrkAβ     330  PPESILYRKFTTESDVWSFGVVLWEIFTYGKQPWYQLSNTEAIDCITQGRELERPRACPPEVYAIMRGCWQREPQQRHSI  409
TrkAγII   665  PPESILYRKFTTESDVWSFGVVLWEIFTYGKQPWYQLSNTEAIDCITQGRELERPRACPPEVYAIMRGCWQREPQQRHSI  744
TrkAδII   641  PPESILYRKFTTESDVWSFGVVLWEIFTYGKQPWYQLSNTEAIDCITQGRELERPRACPPEVYAIMRGCWQREPQQRHSI  720
TrkAεII   510  PPESILYRKFTTESDVWSFGVVLWEIFTYGKQPWYQLSNTEAIDCITQGRELERPRACPPEVYAIMRGCWQREPQQRHSI  589
TrkAζII   400  PPESILYRKFTTESDVWSFGVVLWEIFTYGKQPWYQLSNTEAIDCITQGRELERPRACPPEVYAIMRGCWQREPQQRHSI  479
TrkAηII   315  PPESILYRKFTTESDVWSFGVVLWEIFTYGKQPWYQLSNTEAIDCITQGRELERPRACPPEVYAIMRGCWQREPQQRHSI  394
TrkAθ     281  PPESILYRKFTTESDVWSFGVVLWEIFTYGKQPWYQLSNTEAIDCITQGRELERPRACPPEVYAIMRGCWQREPQQRHSI  360
TrkAκ     232  PPESILYRKFTTESDVWSFGVVLWEIFTYGKQPWYQLSNTEAIDCITQGRELERPRACPPEVYAIMRGCWQREPQQRHSI  311

TrkAII    775  KDVHARLQALAQAPPVYLDVLG  796
TrkAαII   801  KDVHARLQALAQAPPVYLDVLG  822
TrkAβ     410  KDVHARLQALAQAPPVYLDVLG  431
TrkAγII   745  KDVHARLQALAQAPPVYLDVLG  766
TrkAδII   721  KDVHARLQALAQAPPVYLDVLG  742
TrkAεII   590  KDVHARLQALAQAPPVYLDVLG  611
TrkAζII   480  KDVHARLQALAQAPPVYLDVLG  501
TrkAηII   395  KDVHARLQALAQAPPVYLDVLG  416
TrkAθ     361  KDVHARLQALAQAPPVYLDVLG  382
TrkAκ     312  KDVHARLQALAQAPPVYLDVLG  333
```

B

```
TrkAI      1    MLRGGRRGQLGWHSWAAGPGSLLAWLILASAGAAPCPDACCPHGSSGLRCTRDGALDSLHHLPGAENLTELYIENQQHLQ  80
TrkAII     1    MLRGGRRGQLGWHSWAAGPGSLLAWLILASAGAAPCPDACCPHGSSGLRCTRDGALDSLHHLPGAENLTELYIENQQHLQ  80
TrkAIII    1    MLRGGRRGQLGWHSWAAGPGSLLAWLILASAGAAPCPDACCPHGSSGLRCTRDGALDSLHHLPGAENLTELYIENQQHLQ  80
TrkAIV     1    MLRGGRRGQLGWHSWAAGPGSLLAWLILASAGAAPCPDACCPHGSSGLRCTRDGALDSLHHLPGAENLTELYIENQQHLQ  80
TrkAV      1    MLRGGRRGQLGWHSWAAGPGSLLAWLILASAGAAPCPDACCPHGSSGLRCTRDGALDSLHHLPGAENLTELYIENQQHLQ  80
TrkAVI     1    MLRGGRRGQLGWHSWAAGPGSLLAWLILASAGAAPCPDACCPHGSSGLRCTRDGALDSLHHLPGAENLTELYIENQQHLQ  80
TrkAVII    1    MLRGGRRGQLGWHSWAAGPGSLLAWLILASAGAAPCPDACCPHGSSGLRCTRDGALDSLHHLPG----------------  64
TrkAVIII   1    MLRGGRRGQLGWHSWAAGPGSLLAWLILASAGAAPCPDACCPHGSSGLRCTRDGALDSLHHLPGAENLTELYIENQQHLQ  80
TrkAIX     1    MLRGGRRGQLGWHSWAAGPGSLLAWLILASAGAAPCPDACCPHGSSGLRCTRDGALDSLHHLPGAENLTELYIENQQHLQ  80

TrkAI      81   HLELRDLRGLGELRNLTIVKSGLRFVAPDAFHFTPRLSRLNLSFNALESLSWKTVQGLSLQELVLSGNPLHCSCALRWLQ  160
TrkAII     81   HLELRDLRGLGELRNLTIVKSGLRFVAPDAFHFTPRLSRLNLSFNALESLSWKTVQGLSLQELVLSGNPLHCSCALRWLQ  160
TrkAIII    81   HLELRDLRGLGELRNLTIVKSGLRFVAPDAFHFTPRLSRLNLSFNALESLSWKTVQGLSLQELVLSGNPLHCSCALRWLQ  160
TrkAIV     81   HLELRDLRGLGELRNLTIVKSGLRFVAPDAFHFTPRLSRLNLSFNALESLSWKTVQGLSLQELVLSGNPLHCSCALRWLQ  160
TrkAV      81   HLELRDLRGLGELRNLTIVKSGLRFVAPDAFHFTPRLSRLNLSFNALESLSWKTVQGLSLQELVLSGNPLHCSCALRWLQ  160
TrkAVI     81   HLELRDLRGLGELRNLTIVKSGLRFVAPDAFHFTPRLSRLNLSFNALESLSWKTVQGLSLQELVLSGNPLHCSCALRWLQ  160
TrkAVII         --------------------------------------------------------------------------------
TrkAVIII   81   HLELRDLRGLGELRNLTIVKSGLRFVAPDAFHFTPRLSRLNLSFNALESLSWKTVQGLSLQELVLSGNPLHCSCALRWLQ  160
TrkAIX     81   HLELRDLRGLGELRNLTIVKSGLRFVAPDAFHFTPRLSRLNLSFNALESLSWKTVQGLSLQELVLSGNPLHCSCALRWLQ  160

TrkAI      161  RWEEEGLGGVPEQKLQCHGQGPLAHMPNASCGVPTLKVQVPNASVDVGDDVLLRCQVEGRGLEQAGWILTELEQSATVMK  240
TrkAII     161  RWEEEGLGGVPEQKLQCHGQGPLAHMPNASCGVPTLKVQVPNASVDVGDDVLLRCQVEGRGLEQAGWILTELEQSATVMK  240
TrkAIII    161  RWEEEGLGGVPEQKLQCHGQGPLAHMPNASC-------------------------------------------------  191
TrkAIV     161  RWEEEGLGGVPEQKLQCHGQGPLAHMPNASC-------------------------------------------------  191
TrkAV      161  RWEEEGLGGVPEQKLQCHGQGPLAHMPNASC-------------------------------------------------  191
TrkAVI     161  RWEEEGLGGVPEQKLQCHGQGPLAHMPNASCGVPTLKVQVPNASVDVGDDVLLRCQVEGRGLEQAGWILTELEQSATVMK  240
TrkAVII    65   ---------------------------------------------------------------------TELEQSATVMK  76
TrkAVIII   161  RWEEEGLGGVPEQKLQCHGQGPLAHMPNASCGPGAQCLGGCGGRRAAAVPGGGAGPGAGRLDPHRAGAVSHGD-------  233
TrkAIX     161  RWEEEGLGGVPEQKLQCHGQGPLAHMPNASCGPGAQCLGGCGGRRAAAVPGGGAGPGAGRLDPHRAGAVSHGD-------  233
```

```
TrkAI     241  SGGLPSLGLTLANVTSDLNRKNVTCWAENDVGRAEVSVQVNVSFPASVQLHTAVEMHHWCIPFSVDGQPAPSLRWLFNGS  320
TrkAII    241  SGGLPSLGLTLANVTSDLNRKNVTCWAENDVGRAEVSVQVNVSFPASVQLHTAVEMHHWCIPFSVDGQPAPSLRWLFNGS  320
TrkAIII   192  -------------------------------------------VPASVQLHTAVEMHHWCIPFSVDGQPAPSLRWLFNGS  228
TrkAIV    192  -------------------------------------------VPASVQLHTAVEMHHWCIPFSVDGQPAPSLRWLFNGS  228
TrkAV          --------------------------------------------------------------------------------
TrkAVI    241  SGGLPSLGLTLANVTSDLNRKNVTCWAENDVGRAEVSVQVNVSY------------------------------------  284
TrkAVII    77  SGGLPSLGLTLANVTSDLNRKNVTCWAENDVGRAEVSVQVNVSFPASVQLHTAVEMHHWCIPFSVDGQPAPSLRWLFNGS  155
TrkAVIII       --------------------------------------------------------------------------------
TrkAIX         --------------------------------------------------------------------------------

TrkAI     321  VLNETSFIFTEFLEPAANETVRHGCLRLNQPTHVNNGNYTLLAANPFGQASASIMAAFMDNPFEFNPEDPIP------DT  394
TrkAII    321  VLNETSFIFTEFLEPAANETVRHGCLRLNQPTHVNNGNYTLLAANPFGQASASIMAAFMDNPFEFNPEDPIPVSFSPVDT  400
TrkAIII   229  VLNETSFIFTEFLEPAANETVRHGCLRLNQPTHVNNGNYTLLAANPFGQASASIMAAFMDNPFEFNPEDPIP------DT  302
TrkAIV    229  VLNETSFIFTEFLEPAANETVRHGCLRLNQPTHVNNGNYTLLAANPFGQASASIMAAFMDNPFEFNPEDPIPVSFSPVDT  308
TrkAV     192  ------------------------------------------------------------------------------DT  193
TrkAVI         -------------------------------------------------------------------------------T  285
TrkAVII   156  VLNETSFIFTEFLEPAANETVRHGCLRLNQPTHVNNGNYTLLAANPFGQASASIMAAFMDNPFEFNPEDPIPVSFSPVDT  235
TrkAVIII  234  ------------------------------------------------------------------------------DT  235
TrkAIX    234  ------------------------------------------------------------------------VSFSPVDT  241

TrkAI     395  NSTSGDPVEKKDETPFGVSVAVGLAVFACLFLSTLLLVLNKCGRRNKFGINRPAVLAPEDGLAMSLHFMTLGGSSLSPTE  474
TrkAII    401  NSTSGDPVEKKDETPFGVSVAVGLAVFACLFLSTLLLVLNKCGRRNKFGINRPAVLAPEDGLAMSLHFMTLGGSSLSPTE  480
TrkAIII   303  NSTSGDPVEKKDETPFGVSVAVGLAVFACLFLSTLLLVLNKCGRRNKFGINRPAVLAPEDGLAMSLHFMTLGGSSLSPTE  382
TrkAIV    309  NSTSGDPVEKKDETPFGVSVAVGLAVFACLFLSTLLLVLNKCGRRNKFGINRPAVLAPEDGLAMSLHFMTLGGSSLSPTE  388
TrkAV     194  NSTSGDPVEKKDETPFGVSVAVGLAVFACLFLSTLLLVLNKCGRRNKFGINRPAVLAPEDGLAMSLHFMTLGGSSLSPTE  273
TrkAVI    286  NSTSGDPVEKKDETPFGVSVAVGLAVFACLFLSTLLLVLNKCGRRNKFGINRPAVLAPEDGLAMSLHFMTLGGSSLSPTE  365
TrkAVII   236  NSTSGDPVEKKDETPFGVSVAVGLAVFACLFLSTLLLVLNKCGRRNKFGINRPAVLAPEDGLAMSLHFMTLGGSSLSPTE  315
TrkAVIII  236  NSTSGDPVEKKDETPFGVSVAVGLAVFACLFLSTLLLVLNKCGRRNKFGINRPAVLAPEDGLAMSLHFMTLGGSSLSPTE  315
TrkAIX    242  NSTSGDPVEKKDETPFGVSVAVGLAVFACLFLSTLLLVLNKCGRRNKFGINRPAVLAPEDGLAMSLHFMTLGGSSLSPTE  321

TrkAI     475  GKGSGLQGHIIENPQYFSDACVHHIKRRDIVLKWELGEGAFGKVFLAECHNLLPEQDKMLVAVKALKEASESARQDFQRE  554
TrkAII    481  GKGSGLQGHIIENPQYFSDACVHHIKRRDIVLKWELGEGAFGKVFLAECHNLLPEQDKMLVAVKALKEASESARQDFQRE  560
TrkAIII   383  GKGSGLQGHIIENPQYFSDACVHHIKRRDIVLKWELGEGAFGKVFLAECHNLLPEQDKMLVAVKALKEASESARQDFQRE  462
TrkAIV    389  GKGSGLQGHIIENPQYFSDACVHHIKRRDIVLKWELGEGAFGKVFLAECHNLLPEQDKMLVAVKALKEASESARQDFQRE  468
TrkAV     274  GKGSGLQGHIIENPQYFSDACVHHIKRRDIVLKWELGEGAFGKVFLAECHNLLPEQDKMLVAVKALKEASESARQDFQRE  353
TrkAVI    366  GKGSGLQGHIIENPQYFSDACVHHIKRRDIVLKWELGEGAFGKVFLAECHNLLPEQDKMLVAVKALKEASESARQDFQRE  445
TrkAVII   316  GKGSGLQGHIIENPQYFSDACVHHIKRRDIVLKWELGEGAFGKVFLAECHNLLPEQDKMLVAVKALKEASESARQDFQRE  395
TrkAVIII  316  GKGSGLQGHIIENPQYFSDACVHHIKRRDIVLKWELGEGAFGKVFLAECHNLLPEQDKMLVAVKALKEASESARQDFQRE  395
TrkAIX    322  GKGSGLQGHIIENPQYFSDACVHHIKRRDIVLKWELGEGAFGKVFLAECHNLLPEQDKMLVAVKALKEASESARQDFQRE  401

TrkAI     555  AELLTMLQHQHIVRFFGVCTEGRPLLMVFEYMRHGDLNRFLRSHGPDAKLLAGGEDVAPGPLGLGQLLAVASQVAAGMVY  634
TrkAII    561  AELLTMLQHQHIVRFFGVCTEGRPLLMVFEYMRHGDLNRFLRSHGPDAKLLAGGEDVAPGPLGLGQLLAVASQVAAGMVY  640
TrkAIII   463  AELLTMLQHQHIVRFFGVCTEGRPLLMVFEYMRHGDLNRFLRSHGPDAKLLAGGEDVAPGPLGLGQLLAVASQVAAGMVY  542
TrkAIV    469  AELLTMLQHQHIVRFFGVCTEGRPLLMVFEYMRHGDLNRFLRSHGPDAKLLAGGEDVAPGPLGLGQLLAVASQVAAGMVY  548
TrkAV     354  AELLTMLQHQHIVRFFGVCTEGRPLLMVFEYMRHGDLNRFLRSHGPDAKLLAGGEDVAPGPLGLGQLLAVASQVAAGMVY  433
TrkAVI    446  AELLTMLQHQHIVRFFGVCTEGRPLLMVFEYMRHGDLNRFLRSHGPDAKLLAGGEDVAPGPLGLGQLLAVASQVAAGMVY  525
TrkAVII   396  AELLTMLQHQHIVRFFGVCTEGRPLLMVFEYMRHGDLNRFLRSHGPDAKLLAGGEDVAPGPLGLGQLLAVASQVAAGMVY  475
TrkAVIII  396  AELLTMLQHQHIVRFFGVCTEGRPLLMVFEYMRHGDLNRFLRSHGPDAKLLAGGEDVAPGPLGLGQLLAVASQVAAGMVY  475
TrkAIX    402  AELLTMLQHQHIVRFFGVCTEGRPLLMVFEYMRHGDLNRFLRSHGPDAKLLAGGEDVAPGPLGLGQLLAVASQVAAGMVY  481

TrkAI     635  LAGLHFVHRDLATRNCLVGQGLVVKIGDFGMSRDIYSTDYYRVGGRTMLPIRWMPPESILYRKFTTESDVWSFGVVLWEI  714
TrkAII    641  LAGLHFVHRDLATRNCLVGQGLVVKIGDFGMSRDIYSTDYYRVGGRTMLPIRWMPPESILYRKFTTESDVWSFGVVLWEI  720
TrkAIII   543  LAGLHFVHRDLATRNCLVGQGLVVKIGDFGMSRDIYSTDYYRVGGRTMLPIRWMPPESILYRKFTTESDVWSFGVVLWEI  622
TrkAIV    549  LAGLHFVHRDLATRNCLVGQGLVVKIGDFGMSRDIYSTDYYRVGGRTMLPIRWMPPESILYRKFTTESDVWSFGVVLWEI  628
TrkAV     434  LAGLHFVHRDLATRNCLVGQGLVVKIGDFGMSRDIYSTDYYRVGGRTMLPIRWMPPESILYRKFTTESDVWSFGVVLWEI  513
TrkAVI    526  LAGLHFVHRDLATRNCLVGQGLVVKIGDFGMSRDIYSTDYYRVGGRTMLPIRWMPPESILYRKFTTESDVWSFGVVLWEI  605
TrkAVII   476  LAGLHFVHRDLATRNCLVGQGLVVKIGDFGMSRDIYSTDYYRVGGRTMLPIRWMPPESILYRKFTTESDVWSFGVVLWEI  555
TrkAVIII  476  LAGLHFVHRDLATRNCLVGQGLVVKIGDFGMSRDIYSTDYYRVGGRTMLPIRWMPPESILYRKFTTESDVWSFGVVLWEI  555
TrkAIX    482  LAGLHFVHRDLATRNCLVGQGLVVKIGDFGMSRDIYSTDYYRVGGRTMLPIRWMPPESILYRKFTTESDVWSFGVVLWEI  561

TrkAI     715  FTYGKQPWYQLSNTEAIDCITQGRELERPRACPPEVYAIMRGCWQREPQQRHSIKDVHARLQALAQAPPVYLDVLG  790
TrkAII    721  FTYGKQPWYQLSNTEAIDCITQGRELERPRACPPEVYAIMRGCWQREPQQRHSIKDVHARLQALAQAPPVYLDVLG  796
TrkAIII   623  FTYGKQPWYQLSNTEAIDCITQGRELERPRACPPEVYAIMRGCWQREPQQRHSIKDVHARLQALAQAPPVYLDVLG  698
TrkAIV    629  FTYGKQPWYQLSNTEAIDCITQGRELERPRACPPEVYAIMRGCWQREPQQRHSIKDVHARLQALAQAPPVYLDVLG  704
TrkAV     514  FTYGKQPWYQLSNTEAIDCITQGRELERPRACPPEVYAIMRGCWQREPQQRHSIKDVHARLQALAQAPPVYLDVLG  589
TrkAVI    606  FTYGKQPWYQLSNTEAIDCITQGRELERPRACPPEVYAIMRGCWQREPQQRHSIKDVHARLQALAQAPPVYLDVLG  681
TrkAVII   556  FTYGKQPWYQLSNTEAIDCITQGRELERPRACPPEVYAIMRGCWQREPQQRHSIKDVHARLQALAQAPPVYLDVLG  631
TrkAVIII  556  FTYGKQPWYQLSNTEAIDCITQGRELERPRACPPEVYAIMRGCWQREPQQRHSIKDVHARLQALAQAPPVYLDVLG  631
TrkAIX    562  FTYGKQPWYQLSNTEAIDCITQGRELERPRACPPEVYAIMRGCWQREPQQRHSIKDVHARLQALAQAPPVYLDVLG  637
```
