# Supplementary material for: Novel transcripts reveal a complex structure of the human TRKA gene and imply the presence of multiple protein isoforms
Source: BMC Neurosci. 2015 Nov 18;16:78. doi: 10.1186/s12868-015-0215-x (PMC4652384; doi:10.1186/s12868-015-0215-x)
Supplement: Supplementary file 2 — 10.1186/s12868-015-0215-x Primers and cycling conditions used in this study. [file 12868_2015_215_MOESM2_ESM.pdf]

Primers and cycling conditions

RT-PCR analysis
Human tissues

Primer	Primer sequence (5'→3')	Location (exon)	Annealing temperature (°C)	Synthesis time (s)	Nr of cycles	Amplicon size
Forward	CAACTCGGCGCATGAAGGAG	A	59	35	42	727 (A-B-C-D-2-3-4-5); 531 (A-C-D-2-3-4-5); 391 (A-D-2-3-4-5)
Reverse	CGCAGGGCACAAGAACAGTG	5				
Forward	CTTGGTTGGACATCTAAGACCTG	C	61	30	46	416 (C-D-2b-3-4-5); 394 (C-D-2-3-4-5)
Reverse	CGCAGGGCACAAGAACAGTG	5				
Forward	CTAGATCTCGGTGCACAAACTTG	D	62	20	46	896 (D-2-3-4-5-6-7-8); 763 (D-2-3-4-5-6-8); 474 (D-2-3-4-8); 391 (D-7-8); 258 (D-8)
Reverse	GTTGCCGTTGTTGACGTGGGTG	8				
Forward	CTGACAACTGAGGGGAGGAC	E	61	30	40	610 (E-Fc-2-3-4-5); 562 (E-Fb-2-3-4-5); 515 (E-Fa-2-3-3b-4-5); 473 (E-Fa-2-3-3a-4-5); 408 (E-2-3-3b-4-5); 387 (E-Fa-2-3-4-5); 280 (E-2-3-4-5)
Reverse	CGCAGGGCACAAGAACAGTG	5				
Forward	GCTTGGCTGATACTGGCATCTG	1	62	60	42	1005 (1-2-3-4-5-6-7-8); 872 (1-2-3-4-5-6-8); 716 (1-2-3-4-7-8); 583 (1-2-3-4-8); 575 (1d-6b-7-8a); 510 (1c-6c-7-8); 367 (1-8)
Reverse	GTTGCCGTTGTTGACGTGGGTG	8				
Forward	CTCTCTCTCTCTCTCTTGCTGT	G	61	30	40	424 (Ga-2-3-4-5); 392 (Gc-2-3-4-5); 274 (Gb-2-3-4-5)
Reverse	GTTCAGGCACTCCGCCCAGTC	5				
Forward	CTCATTGCTCCTCTCCTCTTTC	2a	61	30	39	1119 (2a-3-4-5)
Reverse	GTTCAGGCACTCCGCCCAGTC	5				
Forward	GGCGGAGTGCCTGAACAGAAG	5	60	70	35	924 (5-6-7-8-9-10-11-12); 791 (5-6-8-9-10-11-12); 648 (5-8-9-10-11-12); 579 (5-6-7-10-11-12); 446 (5-6-10-11-12); 303 (5-10-11-12)
Reverse	GGAGCTGCCACCCAATGTCATG	12				
Forward	AGTTCAACCCCGAGGACCCCATC	8	59	30	33	275 (8-9-10-11-12); 257 (8-10-11-12)
Reverse	GGAGCTGCCACCCAATGTCATG	12				
Forward	GCTTTCTCTCCTCCCTCCTGCTG	10a	61	30	35	444 (10a-11-12); 274 (9a-10-11-12)
Reverse	GGAGCTGCCACCCAATGTCATG	12				
Forward	GCAAAGGCTCTGGGCTCCAAG	12	61	80	34	884 (12-13-14-15-16-17)
Reverse	CTTGATGCTGTGGCGTTGCTG	17				

Mouse tissues

Primer	Primer sequence (5'→3')	Location (exon)	Annealing temperature (°C)	Synthesis time (s)	Nr of cycles	Amplicon size
Forward	ACTTCGTTGATGCTGGCCTGTG	1	61	65	36	1015 (1-2-3-4-5-6-7-8)
Reverse	AGTTCCCGTTGTTGACATGCGTG	8				
Forward	GCATTGTTCCTGTGCCCTGTTC	5	57	65	35	989 (5-6-7-8-9-10-11-12)
Reverse	AAAGAGAACTGCCACCCAGTGTC	12				
Forward	AGTTCCCGTTGTTGACATGCGTG	8	55	45	33	279 (8-9-10-11-12); 261 (8-10-11-12)
Reverse	AAAGAGAACTGCCACCCAGTGTC	12				
Forward	CTATCCCATAAAGGATCATCCTG	10a	57	35	38	496 (10a-11-12); 339 (9a-10-11-12); 236 (9a-10-12)
Reverse	AAAGAGAACTGCCACCCAGTGTC	12				
Forward	CTGGGTGGCAGTTCTCTTTC	12	57	65	32	925 (12-13-14-15-16-17)
Reverse	CGTGCACATCCTTCATGCTGAG	17				

Rat tissues

Primer	Primer sequence (5'→3')	Location (exon)	Annealing temperature (°C)	Synthesis time (s)	Nr of cycles	Amplicon size
Forward	GACTTCGTTGATGCTGGCTTGTG	1	57	30	36	405 (1-2-3-4-5); 258 (1-4-5)
Reverse	CACAATAGGGCACAGGAACAGTG	5				
Forward	AGCAGGAGGATTTGTGTGGTGTG	5	57	60	33	951 (5-6-7-8-9-10-11-12)
Reverse	AAAGAGAACTGCCACCCAGTGTC	12				
Forward	AGTTCCCGTTGTTGACATGCGTG	8	55	45	33	279 (8-9-10-11-12); 261 (8-10-11-12)
Reverse	AAAGAGAACTGCCACCCAGTGTC	12				
Forward	GTCTGGCCTCTGCTTGCTATGAC	10a	59	45	40	707 (10a-11-12); 550 (9a-10-11-12)
Reverse	AAAGAGAACTGCCACCCAGTGTC	12				
Forward	AGTTCAACCCTGAGGACCCCATC	8	57	70	33	1185 (8-9-10-11-12-13-14-15-16-17)
Reverse	CGTGCACATCCTTCATGCTGAG	17				

HPRT (human, mouse, rat)

Primer	Primer sequence (5'→3')		Annealing temperature (°C)	Synthesis time (s)	Nr of cycles
Forward	GATGATGAACCAGGTTATGAC		57	30	30
Reverse	GTCCTTTTCACCAGCAAGCTTG				

5' RACE analysis
Human tissues

1. round of PCR

Primer	Primer sequence (5'→3')	Location (exon)	Annealing temperature (°C)	Synthesis time (s)	Nr of cycles
Forward	CGACTGGAGCACGAGGACACTGA		1) 72; 2) 70; 3) 66	30	1) 5; 2) 5; 3) 25
Reverse	CCACGAAACGGAGACCACTCTTC	3			
Forward	CGACTGGAGCACGAGGACACTGA		1) 72; 2) 70; 3) 64	75	1) 4; 2) 4; 3) 30
Reverse	GGAGCTGCCACCCAATGTCATG	12			

2. round of PCR

Primer	Primer sequence (5'→3')	Location (exon)	Annealing temperature (°C)	Synthesis time (s)	Nr of cycles
Forward	GGACACTGACATGGACTGAAGGAGTA		67	30	34
Reverse	CCCCTCAGATCACGGAGCTCCAGA	2			
Forward	GGACACTGACATGGACTGAAGGAGTA		67	30	34
Reverse	CGGGTCTCCAGATGTGCTGTTAGT	10			
Forward	GGACACTGACATGGACTGAAGGAGTA		65	30	45
Reverse	GCACCGAGATCTAGCAGCCCGCAAC	D			

2. round of PCR (with Phusion polymerase from New England Biolabs)

Primer	Primer sequence (5'→3')	Location (exon)	Annealing temperature (°C)	Synthesis time (s)	Nr of cycles
Forward	GGACACTGACATGGACTGAAGGAGTA		72	20	33
Reverse	AGAAAGGAAGAGGCAGGCAAAGAC	11			

Mouse and rat tissues

1. round of PCR

Primer	Primer sequence (5'→3')	Location (exon)	Annealing temperature (°C)	Synthesis time (s)	Nr of cycles
Forward	CGACTGGAGCACGAGGACACTGA		1) 72; 2) 70; 3) 64	60	1) 4; 2) 4; 3) 30
Reverse	GTGACTGAGCCGAGGGGTGA	3			
Forward	CGACTGGAGCACGAGGACACTGA		1) 72; 2) 70; 3) 64	60	1) 4; 2) 4; 3) 30
Reverse	GAAGTGTAGGGACATGGCCAGC	12			

2. round of PCR

Primer	Primer sequence (5'→3')	Location (exon)	Annealing temperature (°C)	Synthesis time (s)	Nr of cycles
Forward	GGACACTGACATGGACTGAAGGAGTA		65	60	25
Reverse	CACAAAGCGGAGGCCACTCTTCACG	3			
Forward	GGACACTGACATGGACTGAAGGAGTA		65	60	25
Reverse	CCCAGGCCCTGCAGGTCCTCAAAC	2			
Forward	GGACACTGACATGGACTGAAGGAGTA		65	60	25
Reverse	GCAGGGCGGTTGATCCCAAATTTG	12			
Forward	GGACACTGACATGGACTGAAGGAGTA		65	60	25
Reverse	CTCCACTGGGTCTCTTGATGTGCTG	10			

Cloning of TrkA protein isoform-encoding sequences

Primer	Primer sequence (5'→3')	Location (exon)	Annealing temperature (°C)	Synthesis time (s)	Nr of cycles
Forward	CACCATGCTGCGAGGCGGAC	1	1) 54; 2) 57	150	1) 9; 2) 33
Reverse	CTGCTGGGAGCTATGGGGGATG	17			
Forward	CACCATGAAGGAGGCCGCCCTC	A	1) 56; 2) 59	70	1) 4; 2) 40
Reverse	CGCAGGGCACAAGAACAGTG	5			
Forward	CACCATGCAGTTGCGGGCTGCTAG	D	1) 56; 2) 59	70	1) 4; 2) 40
Reverse	GTTCAGGCACTCCGCCCAGTC	5			
Forward	CACCATGCCCAATGCCAGCTGTG	5	1) 56; 2) 59	70	1) 4; 2) 40
Reverse	GTTGCCGTTGTTGACGTGGGTG	8			
Forward	CACCATGCACCACTGGTGCATC	8	1) 56; 2) 59	70	1) 4; 2) 24
Reverse	GCCCAGGACATCCAGGTAGACAG	17			
Forward	CACCATGTCCCTGCATTTCATGAC	12	1) 56; 2) 59	70	1) 4; 2) 40
Reverse	CAGTCCCTGGCCCACTAGACAG	15			
