# Supplementary material for: Novel transcripts reveal a complex structure of the human TRKA gene and imply the presence of multiple protein isoforms
Source: BMC Neurosci. 2015 Nov 18;16:78. doi: 10.1186/s12868-015-0215-x (PMC4652384; doi:10.1186/s12868-015-0215-x)
Supplement: Supplementary file 3 — 10.1186/s12868-015-0215-x GenBank accession numbers of ESTs identified in this study. [file 12868_2015_215_MOESM3_ESM.pdf]

ESTs identified with 5' RACE of human samples	
**Exons**	**Accession nr**
E-2	JZ719179
1b-2	JZ719180
E-2	JZ719181
Gd-2	JZ719182
E-Fa-2	JZ719183
Gb-2	JZ719184
8b-9-10-11	JZ719185
Ga-2	JZ719186
10b-11	JZ719187
8b-10-11	JZ719188
8b-9-10-11	JZ719189
8b-10-11	JZ719190
8b-9-10-11	JZ719191
8b-10-11	JZ719192
10b-11	JZ719193
8c-10-11	JZ719194
Gb-2-3-4-8-10	JZ719195
1a-2-3-4-5-6a-10	JZ719196
1a-2-3-4-5-6a-9-10	JZ719197
1-2	JZ719198
1-2	JZ719199
1a-2	JZ719200
1a	JZ719201
A-D	JZ719202
11a-11	JZ719203
1-10-11	JZ719204
1-8-10-11	JZ719205

ESTs identified with RT-PCR of human samples	
**Exons**	**Accession nr**
A-B-C-D-2-3-4	JZ719206
A-C-D-2-3-4	JZ719207
A-D-2-3-4	JZ719208
C-D-2b-3-4-5	JZ719209
C-D-2-3-4-5	JZ719210
D-2-3-4-5-6-7-8	JZ719211
D-2-3-4-5-6-8	JZ719212
D-2-3-4-8	JZ719213
D-7-8	JZ719214
D-8	JZ719215
E-Fc-2-3-4	JZ719216
E-Fb-2-3-4	JZ719217
E-Fa-2-3-3b-4-5	JZ719218
E-Fa-2-3-3a-4	JZ719219
E-Fa-2-3-4	JZ719220
E-2-3-3b-4-5	JZ719221
E-2-3-4	JZ719222
1-2-3-4-5-6-7-8	JZ719223
1-2-3-4-5-6-8	JZ719224
1-2-3-4-7-8	JZ719225
1-2-3-4-8	JZ719226
1d-6b-7-8a	JZ719227
1c-6c-7-8	JZ719228
1-8	JZ719229
Ga-2-3-4-5	JZ719230
Gc-2-3-4-5	JZ719231
Gb-2-3-4-5	JZ719232
2a-3-4-5	JZ719233
5-6-7-8-9-10-11-12	JZ719234
5-6-7-8-10-11-12	JZ719235
5-6-8-9-10-11-12	JZ719236
5-6-8-10-11-12	JZ719237
5-8-9-10-11-12	JZ719238
5-8-10-11-12	JZ719239
5-6-7-10-11-12	JZ719240
5-6-10-11-12	JZ719241
5-10-11-12	JZ719242
8-9-10-11-12	JZ719243
8-10-11-12	JZ719244
10a-11-12	JZ719245
9a-10-11-12	JZ719246
12-13-14-15-16-17	JZ719247

ESTs identified with 5' RACE of mouse samples	
**Exons**	**Accession nr**
1-2	JZ880805
1-2-3	JZ880806
8d-9-10	JZ880807
10b	JZ880808

ESTs identified with RT-PCR of mouse samples	
**Exons**	**Accession nr**
1-2-3-4-5-6-7-8	JZ719248
5-6-7-8-9-10-11-12	JZ719249
8-9-10-11-12	JZ719250
8-10-11-12	JZ719251
10a-11-12	JZ719252
9a-10-11-12	JZ719253
9a-10-12	JZ719254
12-13-14-15-16-17	JZ719255

ESTs identified with 5' RACE of rat samples	
**Exons**	**Accession nr**
1-2	JZ880809
1-2	JZ880810
1-2	JZ880811
1-2	JZ880812
1-2	JZ880813
1b-2	JZ880814
8d-10	JZ880815
8b-9-10	JZ880816
8b-9-10	JZ880817

ESTs identified with RT-PCR of rat samples	
**Exons**	**Accession nr**
1-2-3-4-5	JZ719256
1-4-5	JZ719257
5-6-7-8-9-10-11-12	JZ719258
8-9-10-11-12	JZ719259
8-10-11-12	JZ719260
10a-11-12	JZ719261
9a-10-11-12	JZ719262
8-9-10-11-12-13-14-15-16-17	JZ719263
